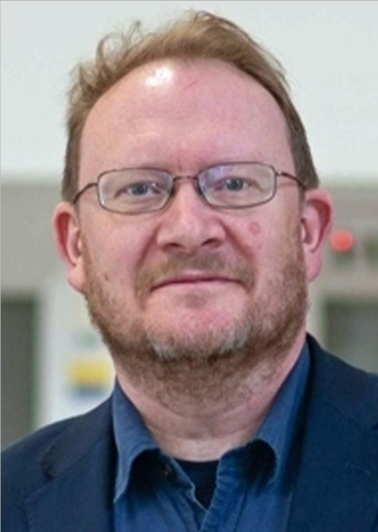# Acid–Base Free Main Group Carbonyl Analogues

**DOI:** 10.1002/anie.202008174

**Published:** 2020-10-19

**Authors:** Ying Kai Loh, Simon Aldridge

**Affiliations:** ^1^ Inorganic Chemistry Laboratory Department of Chemistry University of Oxford South Parks Road Oxford OX1 3QR UK

**Keywords:** carbonyl compounds, main group elements, multiple bonding, oxide transfer, reactivity studies

## Abstract

Main group carbonyl analogues (R_2_E=O) derived from p‐block elements (E=groups 13 to 15) have long been considered as elusive species. Previously, employment of chemical tricks such as acid‐ and base‐stabilization protocols granted access to these transient species in their masked forms. However, electronic and steric effects inevitably perturb their chemical reactivity and distinguish them from classical carbonyl compounds. A new era was marked by the recent isolation of acid–base free main group carbonyl analogues, ranging from a lighter boracarbonyl to the heavier silacarbonyls, phosphacarbonyls and a germacarbonyl. Most importantly, their unperturbed nature elicits exciting new chemistry, spanning the vista from classical organic carbonyl‐type reactions to transition metal‐like oxide ion transfer chemistry. In this Review, we survey the strategies used for the isolation of such systems and document their emerging reactivity profiles, with a view to providing fundamental comparisons both with carbon and transition metal oxo species. This highlights the emerging opportunities for exciting “crossover” reactivity offered by these derivatives of the p‐block elements.

## Graphical abstract

Recently, hitherto elusive acid‐ and base‐free main group carbonyl analogues (R_2_E=O) derived from group 13 to 15 elements have been isolated in crystalline form. Their unperturbed nature elicits exciting new chemistry, spanning the vista from classical organic carbonyl‐type reactions to transition metal‐like oxide ion transfer chemistry.

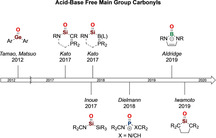

## 1 Introduction

**Scheme 1:**
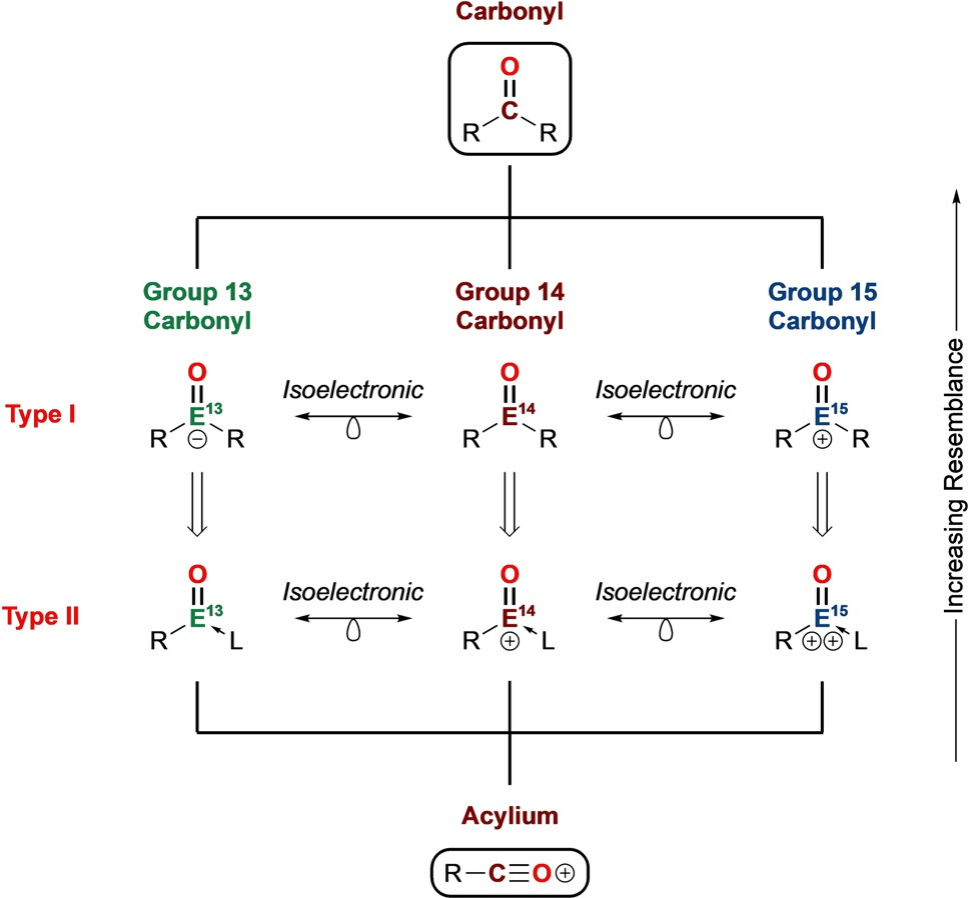
Main group carbonyl analogues of types **I** and **II**.

Carbonyl compounds (R_2_C=O) containing the C=O functional group are ubiquitous in organic chemistry and include aldehydes, ketones, esters, carboxylic acids, amides, ureas etc. (Scheme 1). Their prevalent nature is underscored by the thermodynamic stability of the C=O double bond, which uniquely features σ (392 kJ mol^−1^) and π (399 kJ mol^−1^) components of approximately equal strength.^[1]^ Nevertheless, the charge disparity within the C=O motif (Table 1)^[2]^ induces polarization in the sense C^δ+^−O^δ−^, which, coupled with the sterically open environment, facilitates nucleophilic attack at carbon and electrophilic attack at oxygen. Such reactions can be reversible due to energetically favourable regeneration of the C=O π bond (e.g. addition–elimination reactions at carbon through a tetrahedral intermediate, protonation at oxygen etc.). Hence, the C=O functionality is a unique platform that displays rich chemistry. As such, they represent indispensable chemical building blocks and are cornerstones of organic synthesis.

**Table 1:** Main group p‐block elements, their Pauling electronegativity values and covalent radii [Å] in parentheses.^[2]^ Elements in bold are featured in this Review.

Group 13	Group 14	Group 15	Group 16
**B** **1.88 (0.84)**	**C** **sp^2^ 2.69 (0.73)**	**N** **2.93 (0.71)**	**O** **3.61 (0.66)**
			
**Al** **1.62 (1.21)**	**Si** **2.12 (1.11)**	**P** **2.46 (1.07)**	S 2.64 (1.05)
			
Ga 1.77 (1.22)	**Ge** **2.14 (1.20)**	As 2.25 (1.19)	Se 2.46 (1.20)
			
In 1.63 (1.42)	Sn 2.12 (1.39)	Sb 2.15 (1.39)	Te 2.29 (1.38)
			
Tl 2.00 (1.45)	Pb 2.3 (1.46)	Bi 2.00 (1.48)	Po 2.10 (1.40)

A recent aspiration in synthetic chemistry has been to incorporate p‐block elements into classical organic molecules to construct main group analogues with diverse structural and reactivity profiles.^[3]^ Thus, by substituting carbon with a p‐block element (E=groups 13 to 15), isoelectronic main group carbonyls of the form R_2_E=O (Type **I**) can be conceived (Scheme 1). Alternatively, replacing an R group with a neutral donor L generates main group carbonyls of the form R(L)E=O (Type **II**), which conceptually bear some resemblance to acylium ions. With that said, main group carbonyls of these types (**I** and **II**) containing terminal E=O double bonds are thermodynamically unstable species, in stark contrast to classical carbonyl compounds. The greater electronegativity difference between the main group element and oxygen, coupled with the weaker π overlap, leads to pronounced polarization of the E=O motif, resulting in substantial contribution of the ylidic form E^+^−O^−^. This inability to quench the charge disparity by π bond formation is reminiscent of frustrated Lewis pair (FLP) systems,^[4]^ in which steric constraints impede formation of a σ covalent bond. Hence, the significant “frustration” within the E=O fragment and its sterically exposed position renders them highly reactive and prone to self‐quenching processes (e.g. via head‐to‐tail oligomerization, C−H activation). As such, main group p‐block carbonyl analogues are highly elusive species, often regarded as lab curiosities, and their chemistries have been little developed until recently.

Inspired by the rich chemistry of the C=O functional group, main group chemists have taken up the challenge to synthesize p‐block mimics (Type **I** and **II**). Initial attempts were aimed at their in situ generation and chemical trapping. A major development in this respect was the employment of external acid and base‐stabilization of the E=O functional group, which has enabled the isolation of bottleable main group carbonyl analogues from across the p‐block.^[5]^ However, such methods bring with them inevitable electronic and steric perturbation of the E=O functionality which distinguishes them from the classical C=O functional group.

**Scheme 2:**
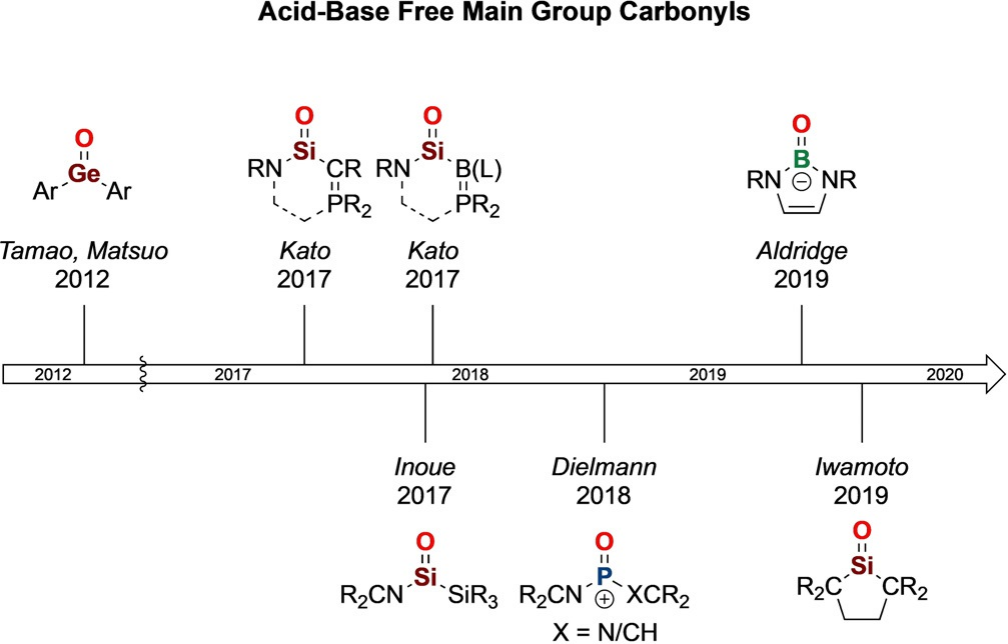
A timeline for the isolation of crystalline acid–base free main group carbonyls.

In 2012, Tamao, Matsuo et al. reported the isolation of the landmark acid–base free germanone (R_2_Ge=O), representing a “genuine” germanium analogue of a ketone (Scheme 2).^[6]^ This compound has subsequently inspired interest in the synthesis of related group 14 systems. In particular, the corresponding silicon analogue has received significant attention due to its position as the lightest “heavy carbonyl”. More than 100 years ago, Kipping attempted to synthesize silanones (R_2_Si=O),^[7]^ producing instead what were later shown to be polysiloxanes (R_2_SiO)_*n*_, and leading to the genesis of a key class of industrial polymers. As such, the isolation of a discrete monomeric silacarbonyl (or silanone, R_2_Si=O) remained elusive for more than 100 years. Kipping's dream was fulfilled in 2017, when Kato et al. reported the breakthrough isolation of room temperature‐stable silacarbonyl species featuring the “free” Si=O motif.^[8]^ Three months later, the groups of Inoue^[9]^ and Kato^[10]^ independently reported stable acyclic silacarbonyls and a bora‐ylide substituted silacarbonyl, respectively. This work inspired other efforts from across the Periodic Table and soon after, in 2018, Dielmann et al. isolated a base‐free phosphacarbonyl analogue, that is, an oxophosphonium ion, [R_2_P=O]^+^, which is isoelectronic with the silacarbonyl.^[11]^ In 2019, we reported the discovery of a lighter carbonyl analogue, i.e. boracarbonyl, in the form of an acid‐free anionic oxoborane [R_2_B=O]^−^, representing an entry point to unperturbed group 13 carbonyl analogues.^[12]^ Within the same month, Iwamoto et al. reported a remarkable dialkylsilanone featuring a “genuine” Si=O double bond.^[13]^

In this Review, we survey main group carbonyl analogues (Type **I** and **II**) from across the p‐block elements (Scheme 1). The discussion is ordered according to position within the Periodic Table and includes (for each group) a summary of the evolution of the field through an overview of preceding acid‐ and base‐stabilized systems, before focusing on recent milestones in the isolation of acid–base free main group carbonyl analogues. We focus on 1) their syntheses, isolation and stabilization strategies, 2) the nature of multiple bonding as reported by structural, spectroscopic and DFT probes, and 3) “unmasked” chemical reactivity and potential future applications. All examples are structurally authenticated unless otherwise stated. Formally triply‐bonded main group monoxide analogous to CO are not included here. For clarity, main group carbonyls are labelled **N** (**N**=number) or **N(X)** to account for simple variation of substituent **X** (**X**=R, LA, L etc.), precursors are labelled **PN** (**P**=precursor) and products of simple addition reactions are labelled **N‐S** (**S**=small molecule).

While traditional Reviews pertaining to main group element chalcogen multiple bonds have tended to focus on elements in a particular group,^[5]^ we hope that the horizontal approach taken by this Review across the p‐block might introduce a new perspective. In particular, recent milestone achievements in taming otherwise highly elusive main group carbonyl analogues in their unperturbed forms present a unique opportunity to use the “free” E=O motif as a basis for lateral comparison of elements across groups 13, 14 and 15 of the p‐block, which might provide new insight into the nature of main group E=O double bonds, and ultimately illuminate similarities and differences with classical C=O chemistry.

## 2 Group 13 Carbonyl Analogues

Group 13 elements have a valence electron count of three, and thus are typically tricoordinate species featuring a trigonal planar geometry. Their reactivity profile is dominated by the presence of a formally vacant p orbital, making them archetypal Lewis acids. Hence, multiply‐bonded species of the type R−E=O featuring strongly Lewis acidic and basic sites adjacent to each other have a marked tendency to oligomerize in head‐to‐tail fashion.^[5a]^ While these oxoboranes (R−B=O) and mono‐alumoxanes (R−Al=O) might be regarded as carbonyl analogues with formal E=O double bonds, their dicoordinate nature and the potential to engage in a further (donor/acceptor) interaction with the terminal oxygen distinguishes them from classical carbonyl compounds. Indeed, the microwave spectrum of gaseous HBO reveals a linear geometry, and a B−O bond length of 1.20 Å, that is confirmed computationally to indicate a B≡O triple bond.^[14]^ Hence, R−E=O species might be thought of as being most closely related to acylium ions (R−C≡O^+^). While “free” R−E=O species are hitherto unknown in the condensed phases, employment of neutral donor ligand L has facilitated isolation of neutral boracarbonyls and alumacarbonyls of the form R(L)E=O (Type **II**). Alternatively, anionic R^−^ ligands have enabled isolation of anionic boracarbonyls and alumacarbonyls of the form [R_2_E=O]^−^ (Type **I**), which are also isoelectronic with carbonyls. Such systematic strategies (employing L or R^−^ ligands) bridge the gap between group 13 elements and carbon by generating isolable group 13 analogues of classical organic functional groups. This notion is perhaps best exemplified by the group 13 alkene analogues, that is, neutral R(L)E=E(L)R and dianionic [R_2_E=ER_2_]^2−^ diborenes^[15]^ and dialumenes.^[16]^ Here, we present the evolution of doubly‐bonded group 13 carbonyl analogues, from their initial isolation as acid–base stabilized entities, to highly reactive dimer‐stabilized alumacarbonyls and a first acid‐free boracarbonyl. A review article by Inoue et al. on multiply‐bonded group 13 element–chalcogen systems documents developments in the field up till 2016, and this material will not be extensively considered here.^[5a]^

### 2.1 Organoboron Oxides

Boron is unique as it is the only main group p‐block element that is lighter than carbon, making boracarbonyls the only lighter analogues of carbonyl compounds. These analogies are also highly pertinent as boron and carbon are neighbours within the second period in the Periodic Table. Boron also has a great affinity for oxygen, affirmed by the thermodynamically strong B−O bond (809 kJ mol^−1^).^[17]^ Its highly oxophilic nature has been widely exploited in organic chemistry to drive industrially important reactions, notably the Suzuki–Miyaura coupling which garnered the Nobel Prize in 2010.^[18]^ This reaction benefits from the use of bench‐stable and non‐toxic organoboron oxides (RB(OR)_2_) as equivalents for otherwise highly reactive carbanions, and their stability can be attributed to the presence of robust B−O linkages. As such, these tricoordinate organoboron oxides containing B−O single bonds are firmly established as an indispensable building block in the organic chemist's toolbox. Although their intrinsic stability is partly attributed to a degree of multiple bonding between boron and oxygen, well‐defined organoboron oxides featuring formal B=O double bonds (i.e. boracarbonyls) have for a long time remained elusive.

In the 1930s, it was discovered that dehydration of boronic acids (RB(OH)_2_) yields not the simple monomeric oxoborane species (R−B=O), but stable boroxines (RBO)_3_ containing a central B_3_O_3_ ring.^[19]^ Oxoboranes were postulated to be generated as short‐lived intermediates that rapidly cyclotrimerize to the corresponding boroxines. Since then, significant effort has been focused on detecting these fleeting species in the gas phase and in low‐temperature matrices.^[5a]^ Pioneering studies by West demonstrated the extreme reactivity of the in situ generated Mes*−B=O molecule through a series of trapping experiments.^[20]^ In this case, kinetic protection from the sole bulky substituent proved insufficient to tame the reactive oxoborane species. While these experiments gave preliminary evidence for their existence, further studies on this interesting compound class are very limited.

### 2.2 Acid‐Stabilized Boracarbonyls

**Scheme 3:**
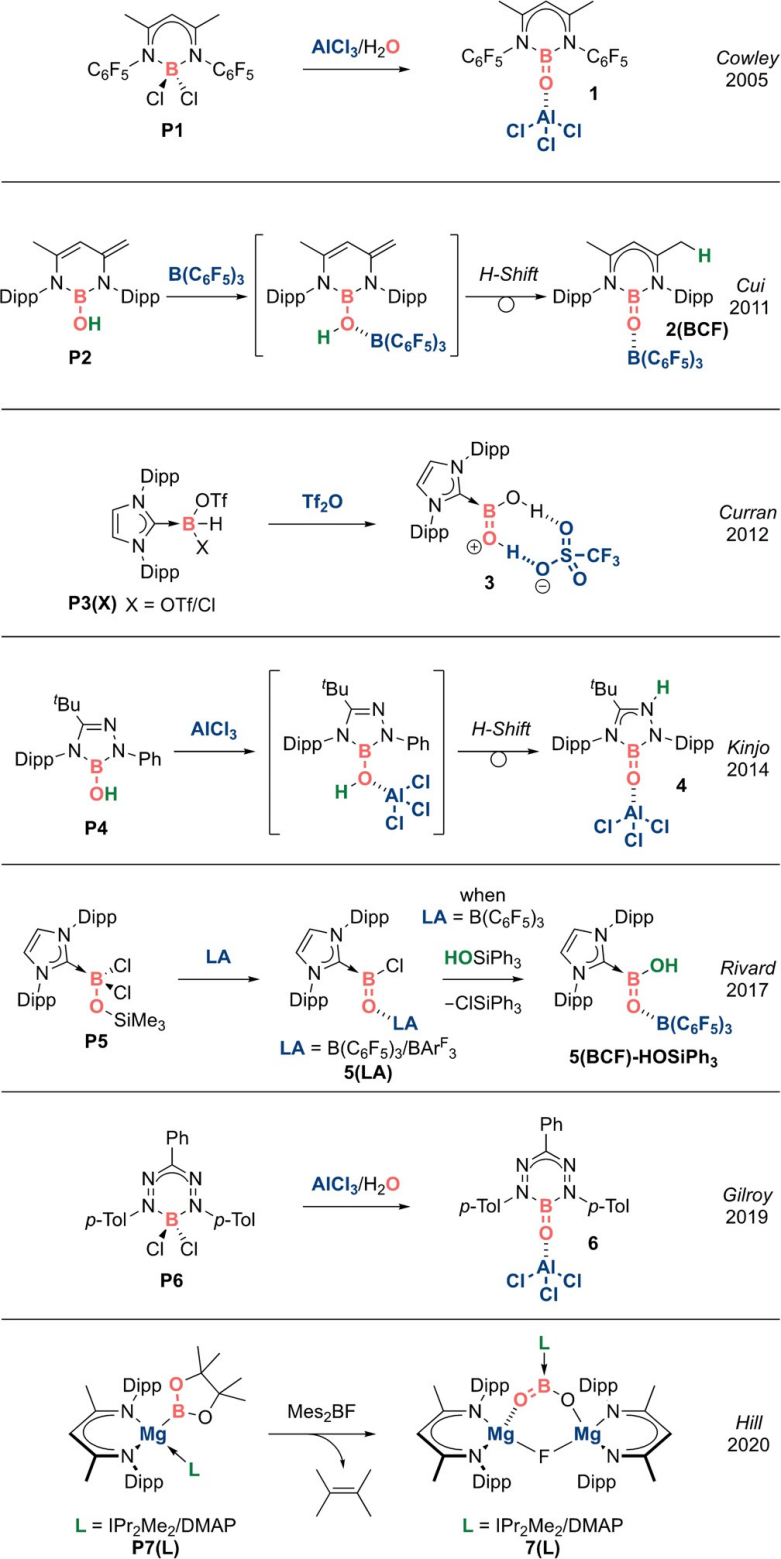
Acid‐stabilized neutral boracarbonyls (Type **II**).

**Neutral analogues: R(L)B=O (Type II)**. A breakthrough in the quest for an isolable oxoborane came in 2005 when Cowley et al. reported the stabilization of a monomeric BO fragment in **1** by simultaneous acid–base coordination (Scheme 3).^[21a]^ This protocol delivers a trigonal planar boron centre featuring a B=O double bond, structurally reminiscent of a carbonyl compound. As such, **1** can be regarded as the first acid‐stabilized boracarbonyl. X‐ray diffraction analysis revealed a short B−O bond of 1.304(2) Å, establishing the notion of a B=O double bond, albeit capped by a Lewis acid. While DFT calculations revealed that AlCl_3_ coordination increases the B−O bond length by only 1.9 % compared to the (hypothetical) acid‐free analogue, the B=O π bond for **1** is significantly lower in energy (HOMO−16) as compared to the acid‐free model (HOMO−6). Hence, while the Lewis acid coordination strategy is effective in isolating a novel boracarbonyl **1**, the inherent electronic perturbation might be expected to moderate the reactivity of the B=O functionality.

In 2011, Cui et al. reported that boron‐based Lewis acids such as B(C_6_F_5_)_3_ can also be employed to stabilize a neutral β‐diketiminate‐derived boracarbonyl, **2(BCF)** (BCF=B(C_6_F_5_)_3_; Scheme 3).^[21b]^ Although the B−OH containing precursor does not spontaneously isomerize to a B=O double bond (which stands in contrast with facile enol–keto tautomerization between C−OH and C=O), O‐to‐C(ligand) proton migration can be induced by Lewis acid coordination at oxygen.

In 2012, Curran et al. reported an NHC‐stabilized dihydroxyborenium cation **3** containing short B−O bonds (mean: 1.3085(5) Å) suggestive of B=O double bond character (Scheme 3).^[21c]^ Hence, **3** can also be viewed as a Brønsted acid‐stabilized neutral boracarboxylic acid.

In 2014, Kinjo et al. reported a Lewis acid‐stabilized neutral boracarbonyl **4** based on a five‐membered 1,2,4,3‐triazaborole framework (Scheme 3).^[21d]^ Interestingly, the amidrazone ligand employed mimics the function of classical β‐diketiminate ligands by inducing 1,4‐oxydrillic proton migration from O‐to‐N(ligand) upon Lewis acid coordination to oxygen.

In 2017, Rivard et al. reported that heating IDipp⋅BCl_2_(OSiMe_3_) in the presence of Lewis acids (LA) such as B(C_6_F_5_)_3_ or BAr^F^_3_ (Ar^F^=3,5‐(CF_3_)_2_C_6_H_3_)_3_ leads to the liberation of ClSiMe_3_ to afford Lewis acid‐stabilized neutral bora‐acyl chlorides **5(LA)** (LA=B(C_6_F_5_)_3_ or BAr^F^_3_) (Scheme 3).^[21e]^ Notably, the B=O π* orbital of **5(BCF)** can be located in the LUMO, in contrast with other systems, in which the corresponding orbital is located at higher energy. This suggests highly electrophilic character, consistent with classical acyl chlorides. However, carbonyl‐like reactions exploiting functionalization of the labile B−Cl bond of **5(BCF)** (to yield B−H or B−R bonds) were unsuccessful, presumably a consequence of steric overcrowding, and electronic perturbation of the reactive B=O unit by the Lewis acid. On the other hand, successful Cl‐for‐OH hydroxylation was achieved using HOSiPh_3_ to generate the corresponding Lewis acid‐stabilized neutral boracarboxylic acid **5(BCF)‐HOSiPh_3_**. DFT analysis revealed that the mechanism for this transformation involves a tetrahedral boron intermediate which collapses via release of ClSiMe_3_, resembling the addition–elimination mechanism of classical nucleophilic acyl substitutions for carbonyl compounds.

In 2019, Gilroy et al. reported a formazanate‐based neutral boracarbonyl **6** stabilized by AlCl_3_ (Scheme 3).^[21f]^ Remarkably, examination of the photoluminescent properties revealed that **6** exhibits a small Stokes shift (50 nm) and has a photoluminescence intensity enhancement of more than 36‐fold (*Φ*_PL_: 36 %), in comparison with **P6**, which has large Stokes shift (174 nm) and was essentially found to be non‐emissive in solution (*Φ*_PL_: <1 %). DFT analysis revealed that **P6** is highly bent in the ground state and perfectly planar in the excited state, whereas the molecular geometry of photoexcited **6** undergoes little structural distortion and resembles its ground state. Hence, it would seem that formation of the exocyclic B=O π bond in **6** significantly improves the rigidity of the system, mitigating non‐radiative decay, thus reducing the Stokes shift and turning on photoluminescence. This work opens up opportunities for main group carbonyls featuring rigid E=O double bonds to be exploited in the design of materials with turn‐on photoluminescence properties.

In 2020, Hill et al. reported the remarkable Lewis acid–base trapping of the highly elusive boron dioxide anion [O=B=O]^−^ to access acid‐stabilized neutral boracarboxylate **7(IPr_2_Me_2_)** and boracarbamate **7(DMAP)** (Scheme 3).^[21g]^ This reactivity is analogous to the chemistry of the isoelectronic CO_2_ molecule, which is commonly employed as a C_1_ source to access carboxylic acid derivatives. The anionic boron dioxide motif in **7(L)** (L=IPr_2_Me_2_/DMAP) is generated via an unusual extrusion of 2,3‐dimethyl‐2‐butene from the Bpin moiety; DFT studies revealed that the release of 2,3‐dimethyl‐2‐butene from [Bpin]^−^ to generate [BO_2_]^−^ is highly energetically favourable (−333 kJ mol^−1^). Interestingly, the [(NHC)BO_2_]^−^ fragment in **7(IPr_2_Me_2_)** can be regarded as the singly deprotonated form of the [(NHC)BO(OH)] fragment found in **5(BCF)‐HOSiPh_3_** and the doubly deprotonated form of the fragment [(NHC)B(OH)_2_]^+^ found in **3**. This notion is in line with the stepwise contraction of the mean B=O lengths within the O‐B‐O motifs from [(NHC)BO_2_]^−^ (mean: 1.3330(17) Å) to [(NHC)BO(OH)] (mean: 1.3325(3) Å) to [(NHC)B(OH)_2_]^+^ (mean: 1.3085(5) Å).

**Scheme 4:**
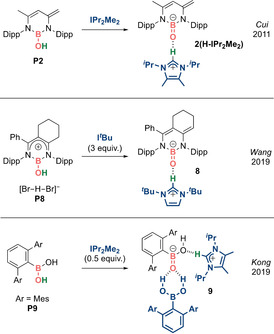
Acid‐stabilized anionic boracarbonyls (Type **I**).

**Anionic analogues: [R_2_B=O]^−^ (Type I)**. In 2011, Cui et al. reported the deprotonation of borinic acid **P2** by IPr_2_Me_2_ (Scheme 4, top).^[21b]^ X‐ray diffraction analysis revealed **2(H‐IPr_2_Me_2_)** to contain a short B−O bond (1.296(3) Å) and a strong hydrogen bonding interaction between the imidazolium moiety and the B=O double bond, representing a Brønsted acid‐stabilized anionic boracarbonyl isoelectronic with urea.

In 2019, Wang et al. reported an imidazolium‐stabilized anionic boracarbonyl **8** supported by a β‐diketiminate ligand featuring a C_6_ fused ring across C1−C2 of the backbone, which was accessed via three‐fold deprotonation of a cationic borinic acid **P8** with I^*t*^Bu (Scheme 4, middle).^[22a]^ More recently, in 2019, Kong et al. reported a unique hydrogen bond‐stabilized boracarboxylic acid anion **9** (Scheme 4, bottom), featuring two sets of hydrogen bond interactions, 1) involving a boronic acid as a bifurcated hydrogen bond donor to the B=O fragment (interaction energies: 160 and 91 kJ mol^−1^); and 2) involving an imidazolium ion acting as a single hydrogen bond donor to the B−OH fragment (interaction energy: 31 kJ mol^−1^).^[22b]^ While hydrogen bonding interactions with C=O fragments have been widely exploited in organocatalysis or molecular recognition, employment of isoelectronic B=O fragments for similar applications can be anticipated.

**Scheme 5:**
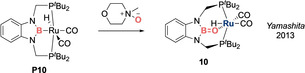
A transition metal‐stabilized boracarbonyl anion.

**A transition metal‐stabilized boracarbonyl**. In 2013, Yamashita et al. reported a diamino boronato ruthenium complex **10**, in which a rigid pincer scaffold enforces an unusually bent B‐O‐Ru angle (93.8(3)°), thereby weakening the O(pπ)−Ru(dπ) interaction and enhancing the B−O π bond (Scheme 5).^[23]^ Accordingly, the B−O bond is short (1.329(6) Å), which, taken together with a WBI of 1.04, indicates a certain degree of B=O double bond character. As such, **10** can be considered as a boracarbonyl anion stabilized by coordination to ruthenium. Carbonyl compounds are well documented to adopt either η^1^ binding mode through oxygen or η^2^ side‐on binding.^[24]^ In the case of **10**, the geometrical imposition of the B=O double bond held by the rigid pincer scaffold hints at the possibility of an η^2^ binding mode. However, the Ru−B bond (2.608(5) Å) is markedly longer than the sum of the covalent radii of Ru and B (2.10 Å) and is therefore more consistent with an η^1^ binding mode through oxygen. This situation hints at the opportunity for fine‐tuning the substituents on boron to bias an η^2^ coordination mode, which is hitherto unknown for B=O fragments. Conversely, one can also envisage harnessing the potential of the highly polarized B=O π bond inherent in the [(R_2_N)_2_BO]^−^ unit to design strongly donating η^1^ O‐donor ligands.

### 2.3 An Acid‐Free Boracarbonyl: The First Lighter Carbonyl

**Figure 1:**
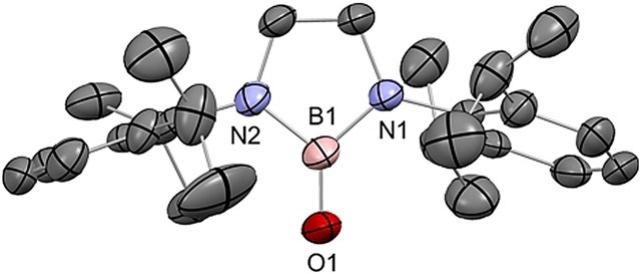
Solid‐state structure of **11**. For clarity, [K(2.2.2‐crypt)]^+^ and hydrogen atoms are omitted. Thermal ellipsoids set at 50 % probability.

**Scheme 6:**
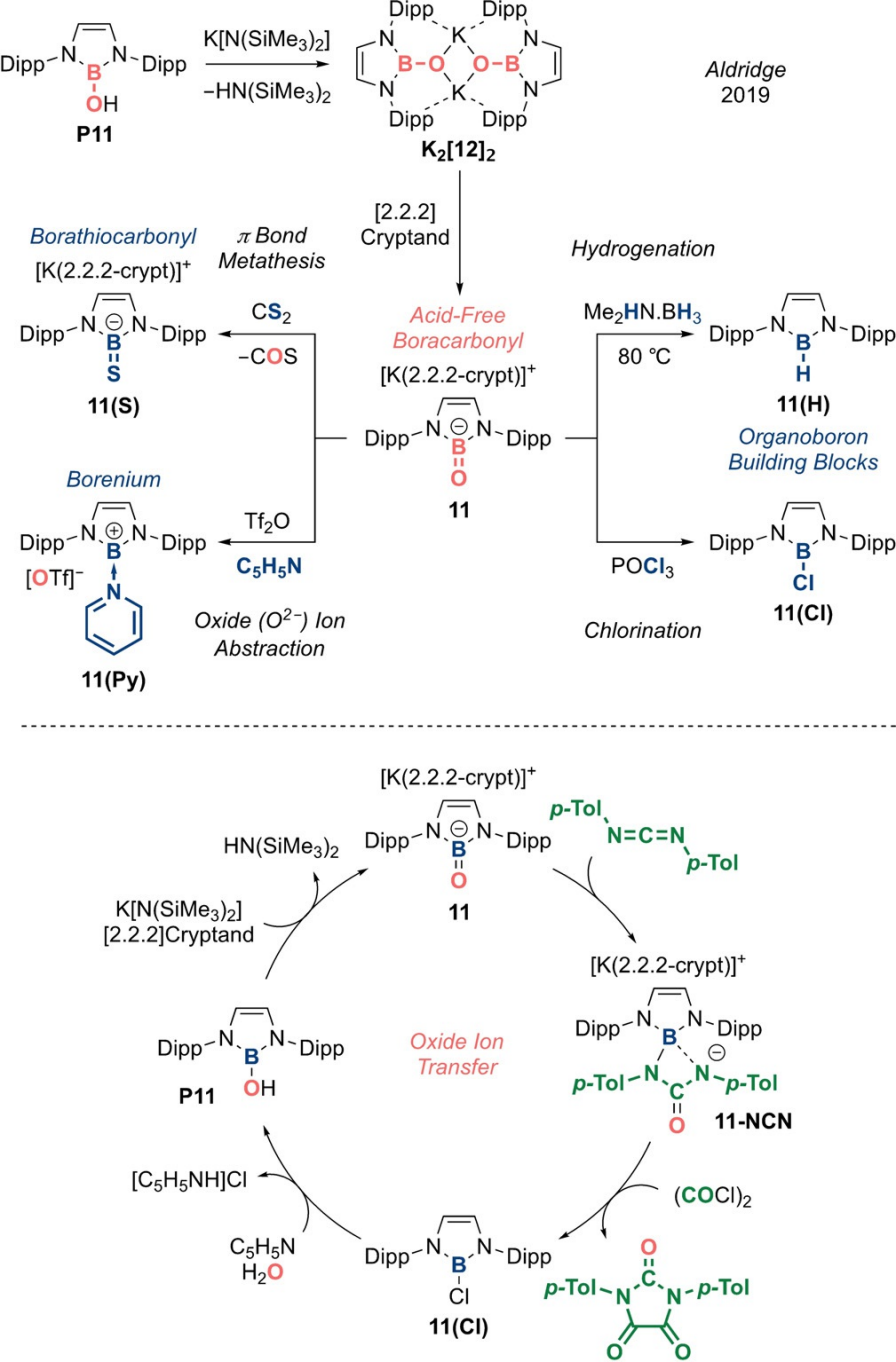
Acid‐free boracarbonyl **11** (Type **I**) and its reactivity as an oxide ion transfer agent.

In 2019, we reported an acid‐free boracarbonyl **11** (Type **I**), representing the first lighter carbonyl analogue (Scheme 6, top).^[12a]^ Its unique stability can be attributed to two factors, 1) the 6π aromatic diazaborole framework which reduces the inherent Lewis acidity of the boron centre, and 2) encapsulation of the potassium counter‐ion by [2.2.2]cryptand, freeing it from the coordination capabilities of the strongly basic oxygen atom. Starting from the borinic acid **P11**, deprotonation with K[N(SiMe_3_)_2_] gave rise to dimer‐stabilized boracarbonyl **K_2_[12]_2_**, and subsequent sequestration by [2.2.2]cryptand allowed access to the stable monomeric boracarbonyl anion **11**. X‐ray diffraction analysis confirmed encapsulation of the potassium ion by [2.2.2]cryptand, thereby distancing it from the terminal oxygen atom (O−K: 5.919(6) Å; Figure 1). The B−O bond (1.273(8) Å) is very short compared to acid‐stabilized boracarbonyls (1.287(4)–1.329(6) Å), reflecting the effect of acid liberation. It is also shorter by 0.10 Å (ca. 8 %) compared to borinic acid **P11**, suggesting enhanced O‐to‐B π donation on deprotonation. DFT analysis revealed the WBI of the B=O bond to be 1.40, that is, considerably greater than acid‐stabilized boracarbonyls (1.04–1.21). NPA charges (B: +0.99, O: −1.03) suggest the presence of a strong ionic component within the B=O motif. As compared with the acid‐protected analogues, the B=O π bond of **11** (HOMO−2) is more energetically accessible, which, coupled with its sterically exposed nature, hints at high levels of reactivity across the unperturbed B=O double bond.

With an acid‐free boracarbonyl compound in hand, the possibilities for carbonyl‐like reactivity of **11** were then probed (Scheme 6, top). Treatment with CS_2_ leads to facile π bond metathesis to afford borathiocarbonyl **11(S)** with concomitant evolution of gaseous COS. X‐ray diffraction analysis revealed **11(S)** to contain a terminal B=S double bond (1.774(1) Å). This represents the first anionic thioxoborane isoelectronic with thiocarbonyls (cationic and neutral thioxoboranes having been reported previously). On the other hand, exhaustive hydrogenation of the B=O double bond with Me_2_HN⋅BH_3_ as a mild hydride source affords the corresponding hydroborane **11(H)** (akin to carbonyl hydrogenation to an alkane). This contrasts with the lack of reactivity observed for Lewis acid‐stabilized bora‐acyl choride **5(BCF)** towards the relatively strong hydride source K[HB(^*s*^Bu)_3_] and underpins the non‐innocent role of the Lewis acid in altering the electronic and steric environment around the B=O fragment. Facile chlorination of **11** can also be achieved with POCl_3_ as the chloride source to afford the corresponding chloroborane **11(Cl)**. Most remarkably, treating **11** with Tf_2_O in the presence of pyridine results in complete abstraction of the oxide ion (O^2−^), to afford an electrophilic borenium cation **11(Py)** stabilized by pyridine.

Lastly, boracarbonyl **11** can also take on the role of an oxide ion transfer agent to an organic substrate, in a similar fashion to the nitrogen transfer exhibited by the isoelectronic nitrene (NHI)_2_P=N. Employing (*p*‐Tol)N=C=N(*p*‐Tol) as the substrate resulted in its insertion into the B=O double bond of **11** to form **11‐NCN** (Scheme 6, bottom). Subsequently, addition of (COCl)_2_ induces release of the O‐functionalized substrate from boron to furnish a urea derivative. Finally, the synthetic cycle could be closed by simple hydrolysis of the chloroborane **11(Cl)** to restore the B−O bond, and borinic acid **P11** can subsequently undergo a deprotonation/sequestration sequence to regenerate **11**. Overall, the boron centre acts as a platform for oxide transfer, mimicking the activity of transition metals.

While B−O bonds are traditionally regarded as thermodynamic sinks and are widely exploited to drive industrially important chemical transformations such as the Suzuki reaction, chemical recycling of the resulting B−O bonds is challenging, and generally involves the use of harsh conditions and reagents. This work demonstrates that the reactivity enhancement for classical doubly‐bonded carbonyl compounds (cf. inert C−O bonds in ethers vs. more reactive C=O double bonds in carbonyls) can be extended to boracarbonyl **11**, in effect facilitating facile cleavage of robust B−O single bonds by exploiting the more reactive terminal B=O double bond, opening new avenues for reversing B−O bond formation under mild conditions.

**Scheme 7:**
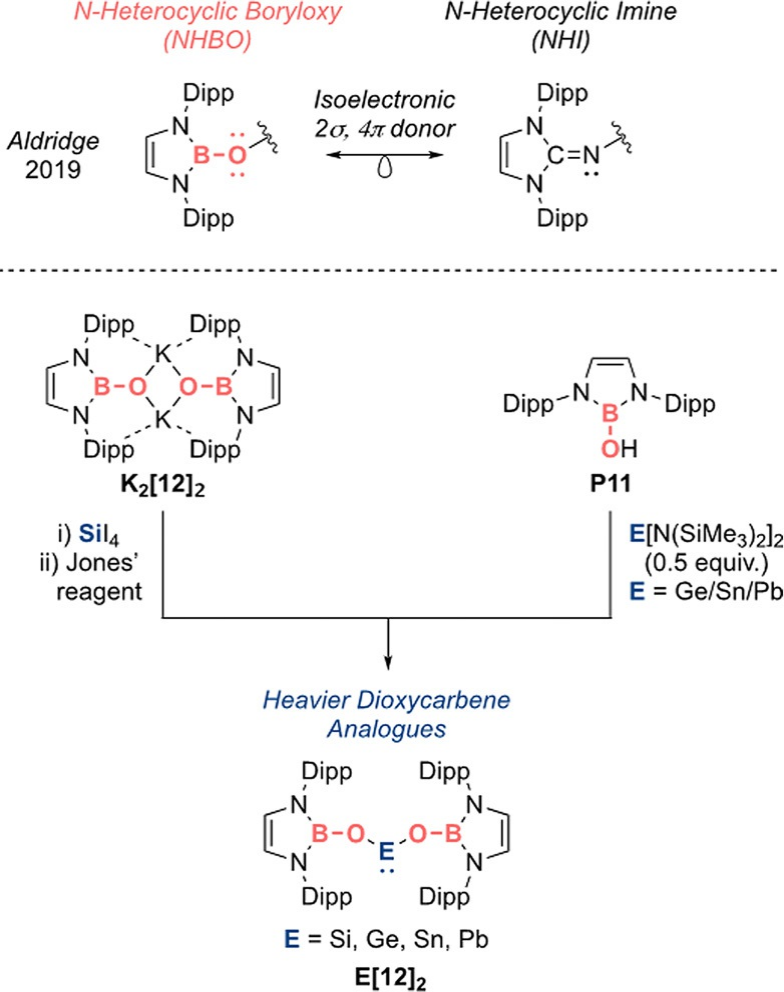
Use of the NHBO ligand as a strong O‐donor to stabilize a series of heavier dioxycarbene analogues.

Classical carbonyl compounds are only weakly basic at the oxygen atom, whereas the isoelectronic boron analogue is anionic, which, coupled with the potent basicity of its oxygen atom, should make them versatile ligands. Indeed, as was demonstrated in another report, the acid‐free boracarbonyl **11** can additionally assume the role of an O‐based ligand (Scheme 7, top).^[12b]^ Thus this new class of N‐heterocyclic boryloxy (NHBO) ligand features a strongly nucleophilic N‐heterocyclic boryl (NHB) unit^[25]^ as the O‐bound substituent and is isoelectronic with the well‐known N‐heterocyclic imine (NHI) ligand.^[26]^ Hence it can be anticipated to possess similar strong 2σ, 4π donor abilities, in addition to a demanding steric profile—two attributes that are absent in classical O‐based ligands. For instance, alkoxides (RO^−^) lack suitable steric protection in the vicinity of the metal centre. Although aryloxides (ArO^−^) with *ortho*‐substitution can screen the metal centre, their good leaving group abilities render them weakly binding ligands. In main group chemistry, ligands that possess simultaneously strong donor and huge steric profile are key to the stabilization of highly reactive low‐valent and low‐coordinate species. Indeed, DFT analysis revealed stronger σ and π donor properties for the NHBO ligand in comparison to other O‐donors (e.g. [{(Me_3_Si)_2_HC}_2_BO]^−^, [(2,6‐Dipp_2_C_6_H_3_)O]^−^)—although not as strong as the donor properties of the N‐based NHI family.

As a proof of concept, we employed the NHBO to tame the first acyclic dicoordinate dioxysilylene **Si[12]_2_** and its heavier congeners **E[12]_2_** (E=Ge, Sn, Pb), thereby completing the series of stable heavy dioxycarbenes (Scheme 7, bottom). **Si[12]_2_** was synthesized by treating potassiated NHBO ligand **K_2_[12]_2_** with SiI_4_ followed by reduction with Jones’ reagent, [(Nacnac)Mg]_2_ (Nacnac=HC(MeCMesN)_2_), and the heavier congeners were synthesized by treating protio‐ligand **P11** with E[N(SiMe_3_)_2_]_2_ (E=Ge, Sn, Pb). Hence, based on the unquenched basicity of the terminal oxygen atom in the acid‐free boracarbonyl, this new class of NHBO ligand offers access to other thermodynamically robust oxy‐stabilized main group systems.

### 2.4 Organoaluminium Oxides

Among the group 13 elements, aluminium is characterized by its highly electropositive nature (Table 1). It also has a noticeably larger atomic size than boron (ca. 44 % larger), thus it tends to adopt coordination numbers above three. Hence, tricoordinate organoaluminium species containing well‐defined Al−O fragments are extremely rare.

Monoalumoxanes (R−Al=O) are the monomeric units of alumoxanes (RAlO)_*n*_. The simplest derivative, methylalumoxane (MeAlO)_*n*_ or MAO, has significant industrial importance as a catalyst activator in olefin polymerization. However, exact details of its structural composition are not definitively known. In 1997, Power et al. attempted to generate a monoalumoxane by employing the much bulkier Mes* substituent.^[27]^ However, this kinetic stabilization approach proved insufficient to circumvent head‐to‐tail oligomerization, and tetrameric (Mes*AlO)_4_—featuring an eight‐membered Al_4_O_4_ ring—was isolated instead. It is noteworthy to compare with West's earlier studies of the lighter homologue Mes*BO, which forms a dimer, hinting at the greater challenge associated with the quest for an isolable monoalumoxane.

Renewed interest in aluminium chemistry has been partly due to the discovery by Aldridge and Goicoechea in 2018 of a new class of low‐valent anionic organoaluminium species featuring a nucleophilic Al^I^ centre that is isoelectronic with carbenes.[^28a^, ^28b^] This report was subsequently followed by related compounds from the groups of Coles, Hill, Yamashita, Kinjo and Harder.[^28c^, ^28d^, ^28e^, ^28f^, ^28g^] The umpolung character of this class of Al^I^ compound has enabled access to unusual organoaluminium compounds previously inaccessible via traditional methods, including alumacarbonyls. Here we survey the literature on these isolable aluminium analogues of carbonyl compounds and focus on the most recent examples and their reactivity studies.

### 2.5 An Acid–Base Stabilized Alumacarbonyl

**Scheme 8:**
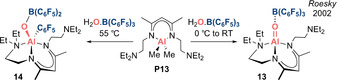
Acid–base stabilized neutral alumacarbonyl **13** (Type **II**).

**Neutral analogue: R(L)Al=O (Type II)**. In 2002, Roesky et al. reported the discovery of an isolable monoalumoxane, which can also be regarded as the first acid–base stabilized alumacarbonyl **13** (Scheme 8).^[29]^ The Al−O bond (1.659(3) Å) is the shortest for any tetracoordinate Al−O fragment, hinting at multiple bond character. It is also interesting to compare the B−O distance in the Al=O⋅⋅⋅B(C_6_F_5_)_3_ unit (1.444(3) Å), which is noticeably shorter than the corresponding distances in B(C_6_F_5_)_3_‐stabilized boracarbonyls, (**2(BCF)**, **5(BCF)**, **5(BCF)‐HOSiPh_3_**: 1.484(3)–1.518(3) Å). The suggestion that the Al=O⋅⋅⋅B(C_6_F_5_)_3_ fragment features a stronger B−O interaction is consistent with the idea of weaker Al=O multiple bonding and a dominant Al^+^−O^−^ form. The consequent electrophilicity of the aluminium centre presumably underpins the 1,3‐migration of the C_6_F_5_ group from boron to aluminium observed at elevated temperature.

### 2.6 Dimer‐Stabilized Alumacarbonyls

**Scheme 9:**
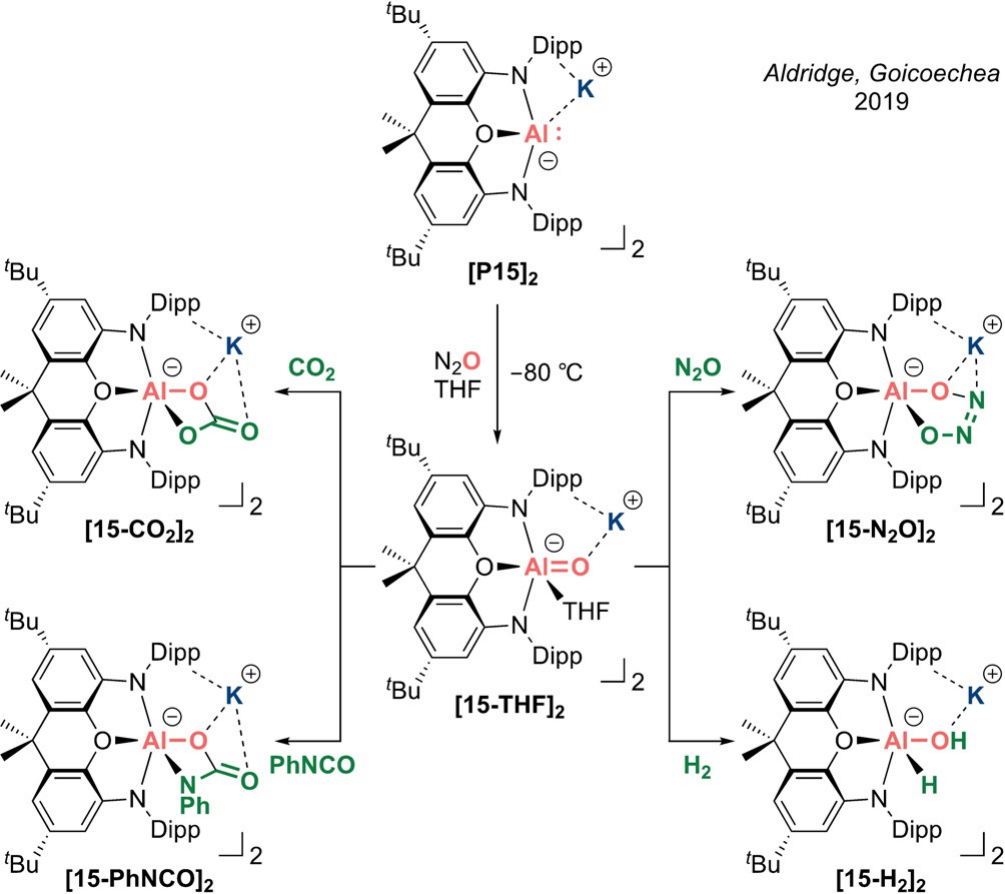
Dimer‐stabilized alumacarbonyl **[15‐THF]_2_** (Type **I**) and its reactivity.

In 2019, Aldridge, Goicoechea et al. reported oxidation of the nucleophilic aluminyl anion **[P15]_2_** to generate an extremely reactive alumacarbonyl anion **[15]_2_**, which can be isolated as a stable THF complex **[15‐THF]_2_** (Scheme 9).^[30a]^ This represents a new approach to form Al−O linkages by exploiting the strong reducing nature of the aluminyl anion and high oxophilicity of aluminium, to drive facile O‐atom abstraction from simple O‐containing small molecules. Treating the aluminyl anion **[P15]_2_** with CO_2_, PhNCO and N_2_O leads to swift uptake of two molecules of the substrate to form anionic Al‐bound carbonate **[15‐CO_2_]_2_**, carbamate **[15‐PhNCO]_2_** and *cis*‐hyponitrite **[15‐N_2_O]_2_**, respectively. A two‐step mechanism was proposed whereby initial O‐atom abstraction generates a common Al=O intermediate **[15]_2_**, which subsequently reacts with another equivalent of CO_2_ or PhNCO to form [2+2] cycloaddition products, while N_2_O (which is a 1,3‐dipolarophile) forms a [3+2] cycloaddition product.

The intrinsic preference for heteroallenes to react with the intermediate alumacarbonyl anion **[15]_2_** while leaving **[P15]_2_** untouched suggests that the Al=O moiety possesses a very high degree of reactivity. Accordingly, while the elusive **[15]_2_** cannot be detected even when carrying out the reactions at −80 °C, changing the solvent to THF allows for the isolation of **[15‐THF]_2_** from the reaction with N_2_O at low temperatures (Scheme 9). X‐ray diffraction analysis revealed that the nominally five‐coordinate aluminium centre adopts a distorted trigonal bipyridamidal geometry, with the oxide and the ligand oxygen assuming apical positions, and the nitrogen donor atoms and THF occupying equatorial positions. Thus, its stability can be attributed to THF occupation of the remaining sterically and electronically exposed vacant p orbital in the equatorial direction, which compensates for the reduced N‐to‐Al π donation, due to the significant distortion imposed by the puckering of the bis(amino)dimethylxanthene ligand. Strikingly, the Al−O bonds (mean: 1.6763(12) Å) are shorter than those found in the [(Nacnac)Al(Me)OLi]_3_ trimer (1.698(1) Å) which contains tetracoordinate aluminum centres, presumably reflecting the considerably weaker Al−O⋅⋅⋅K interactions (cf. Al−O⋅⋅⋅Li). Hence, **[15‐THF]_2_** can be regarded as a dimeric THF‐trapped alumacarbonyl anion. DFT analysis revealed that the model anionic fragment in (monomeric) **15‐THF** features an Al−O distance which is only circa 1 % shorter than in crystallographically determined (dimeric) **[15‐THF]_2_**, giving further evidence that the potassium ions have a minor effect on the Al=O fragment. The WBI value of 0.64 for the Al=O bond and NPA charges of Al (+2.07) and O (−1.52) suggest that the short Al−O bond is due largely to electrostatic interactions, with a minor contribution from the Al=O π component.

In spite of featuring a strongly Lewis acidic aluminium centre adjacent to a strongly Lewis basic oxygen, the inability to quench by π bond formation potentially generates significant chemical “frustration”. Accordingly, exposure of **[15‐THF]_2_** to H_2_ affords the 1,2‐addition product **[15‐H_2_]_2_** and substantiates the hypothesis that highly polarized E=O bonds within main group carbonyl analogues can exhibit FLP‐like reactivity (Scheme 9).

**Scheme 10:**
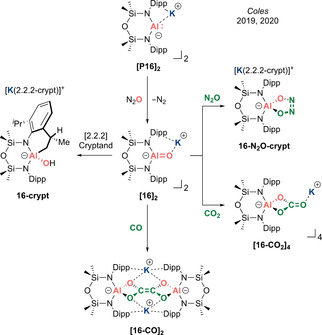
Dimer‐stabilized alumacarbonyl **[16]_2_** (Type **I**) and its reactivity.

In 2019, a near‐simultaneous report by Coles et al. described the isolation of a remarkably stable planar tricoordinate alumacarbonyl anion **[16]_2_** (Type **I**).^[30b]^ This was achieved by exposing dicoordinate potassium aluminyl **[P16]_2_** to N_2_O at room temperature (Scheme 10). X‐ray diffraction analysis confirmed a dimeric structure with the bridging potassium ions sandwiched between the arene π systems. It is noteworthy that this dimeric form resembles the lighter boron homologue of the NHBO potassium dimer **K_2_[12]_2_**. The most dominant feature is the three‐coordinate aluminium centre, with an extremely short Al−O bond (mean: 1.6362(14) Å)—noticeably shorter than Roesky's four‐coordinate Lewis acid‐stabilized neutral alumacarbonyl (1.659(3) Å) and significantly shorter than five‐coordinate THF‐trapped **[15‐THF]_2_** (mean: 1.6763(12) Å), indicating the sensitivity of the Al−O bond to the coordination number at the aluminium centre. The significantly more stable nature of **[16]_2_** derives from the planarization of the aluminium centre, enabling efficient π donation from the nitrogen atoms and oxide ion to quench its Lewis acidity. DFT analysis on the model anionic fragment in (monomeric) **16** revealed that the Al−O bond is only 0.5 % shorter than in experimentally determined (dimeric) **[16]_2_**, suggesting the minimal influence of the Al−O⋅⋅⋅K interactions, hence giving credence to the notion of **[16]_2_** being considered a dimeric alumacarbonyl anion approaching its acid‐free form. The WBI of 0.86 for the Al=O fragment of **16** is greater than that for **15‐THF** (0.64), indicating a greater degree of covalency in the Al=O π bond. However, NPA charges of Al (+2.03) and O (−1.46) revealed that the Al−O interaction still possesses dominant ionic character. The Al=O π bond can be located in the HOMO−1.

While DFT analysis might suggest only minor influence of the potassium ions in **[16]_2_**, complete sequestration by [2.2.2]cryptand “unmasks” the true nature of the alumacarbonyl moiety and results in intramolecular C(sp^3^)−H activation of the proximal methyl group on the flanking Dipp substituent across the Al=O bond to form **16‐crypt** (Scheme 10). Freeing the flanking Dipp groups from η^6^ coordination with the potassium ions presumably facilitates free rotation, positioning the Me group close to the newly exposed Al=O unit. Hence, it would appear that the influence of the potassium ions in maintaining a degree of structural rigidity in the dimeric form contributes crucially towards the overall stability of **[16]_2_**.

Analogous to **[15‐THF]_2_**, **[16]_2_** also undergoes a [2+3] cycloaddition with N_2_O and [2+2] cycloaddition with CO_2_ to afford **16‐N_2_O‐crypt** and **[16‐CO_2_]_4_**, respectively, as confirmed by X‐ray diffraction analysis (Scheme 10). More recently, Coles et al. reported the remarkable reactivity of **[16]_2_** with two molecules of CO to afford a new ethene–tetraolate [C_2_O_4_]^4−^ ligand held within the Al_2_K_2_ pocket of **[16‐CO]_2_**.^[30c]^ X‐ray diffraction analysis revealed a central C=C double bond (1.338(2) Å) and C−O single bonds (mean: 1.3842(15) Å), distinguishing it from the common oxalate [C_2_O_4_]^2−^ ligand and indicating a formulation more consistent with the further reduced ethene–tetraolate tetra‐anion [C_2_O_4_]^4−^. This work represents proof of concept that an alumacarbonyl anion can mimic certain aspects of transition metal chemistry to promote the elaboration of C_1_ sources via the construction of new C−C bonds to access more complex molecules.

## 3 Heavier Group 14 Carbonyl Analogues

Like carbon, the heavier group 14 elements have four valence electrons. Hence, heavier group 14 carbonyls (especially those of silicon) have received considerable attention as their valence isoelectronic relationship offers direct comparison with classical carbonyl compounds. Since the introduction of donor–acceptor stabilization by Driess et al. in 2007, examples of isolable acid–base stabilized silacarbonyls have expanded rapidly. Silacarbonyls can also attain stability within the coordination spheres of transition metals. In sharp contrast, the heavier germacarbonyl analogues are significantly less explored, and isolable stanna‐ and plumbacarbonyls are hitherto unknown. Here, we present the evolution of the group 14 carbonyl analogues from stabilized entities to acid–base free species and their ensuing reactivity studies. Notably, a review article by Driess et al. on heavier group 14 carbonyl analogues documents the developments of the field up to 2013.^[5b]^

### 3.1 Organosilicon Oxides

Silicon and oxygen are the two most abundant elements in the Earth's crust—Si (28 %) and O (46 %), and their great affinity for each other is reflected in the exceptionally strong Si−O σ bond (501 kJ mol^−1^).^[1]^ Hence, robust materials made of silicon oxides (e.g. glass, polymers and semiconductors) play an integral role in our everyday lives. Despite this, on the molecular level, organosilicon oxide chemistry is still in its infancy. Organic chemists primarily exploit the strong oxophilicity and substantial steric bulk of tetracoordinate organosilanes to act as protecting groups. From a fundamental perspective, an organosilicon oxide featuring a higher bond order is of considerable interest, and silacarbonyls featuring Si=O double bonds are regarded as the lightest “heavy carbonyl” compounds. However, theoretical studies predict that the strength of the Si=O π bond (245 kJ mol^−1^) is only half that of the corresponding σ bond, in sharp contrast with carbonyl compounds.^[1]^ As such, the isolation of discrete monomeric silanones analogous with classical ketones is extremely challenging—reflected in the fact that they remained elusive for more than 100 years.

### 3.2 Acid–Base Stabilized Silacarbonyls

**Scheme 11:**
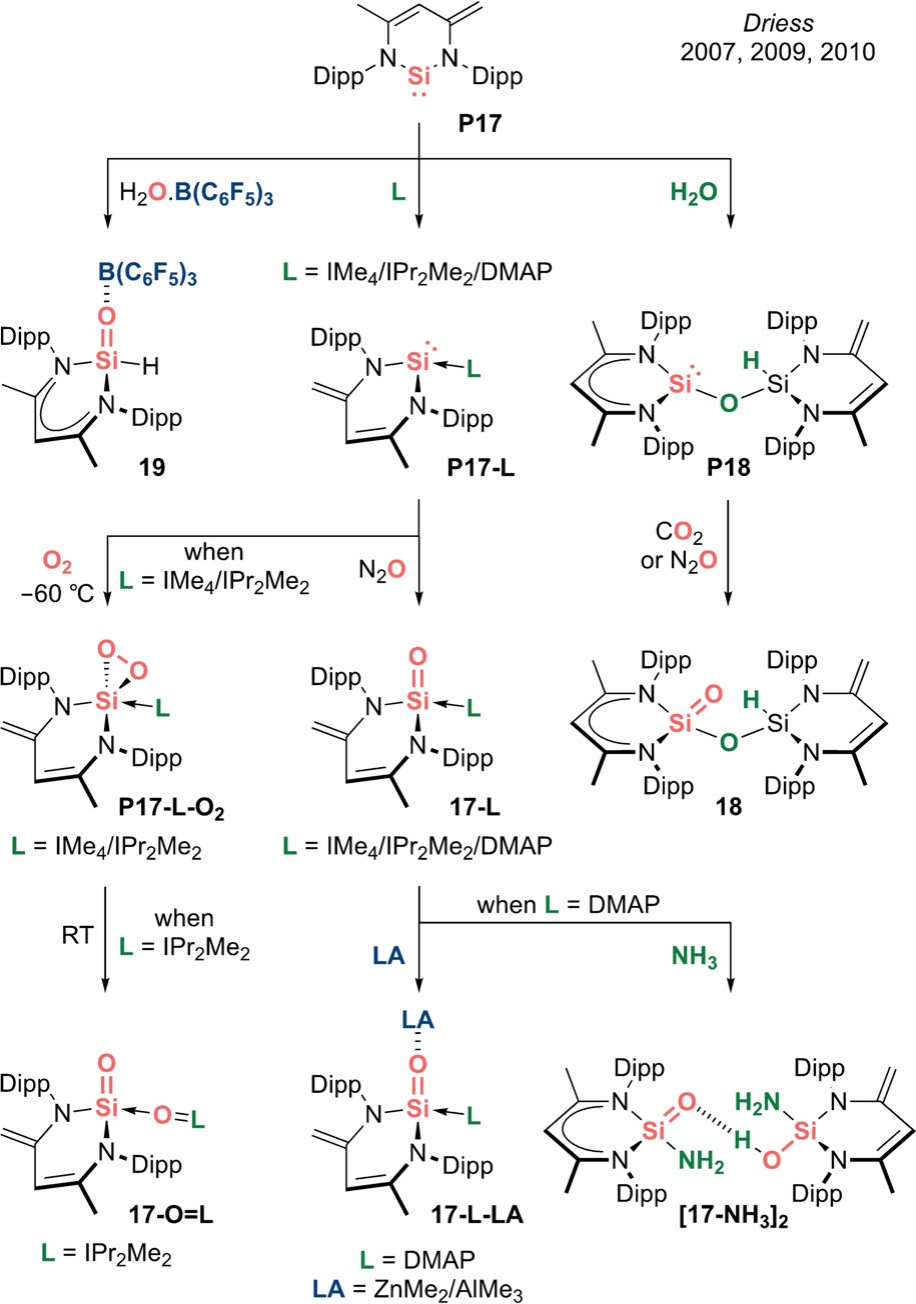
Driess’ acid–base stabilized silacarbonyls (Type **I**).

**Neutral analogues: R_2_Si=O (Type I)**. In 2007, seminal work by Driess et al. introduced the donor–acceptor strategy to tame the highly reactive Si=O moiety and generate a variety of bottleable silacarbonyl species (Scheme 11). The first example to be reported was a β‐diketiminate‐supported silaformaldehyde **19** capped by B(C_6_F_5_)_3_, in which a short Si=O double bond (1.552(2) Å) is identified.^[31a]^ Other notable derivatives include acid–base stabilized silaureas **17‐L‐LA** (L=DMAP; LA=ZnMe_2_/AlMe_3_) and a sila‐amide **[17‐NH_3_]_2_**.[^31b^, ^31c^] The Driess group further discovered that silaureas **17‐L** (L=IMe_4_/IPr_2_Me_2_/DMAP), **17‐O=IPr_2_Me_2_** and silaester **18** can be stable in the absence of acid protection.[^31d^, ^31e^, ^31f^, ^31g^] It is remarkable that these silacarbonyl compounds could all be derived from the same silylene precursor **P17**.

**Scheme 12:**
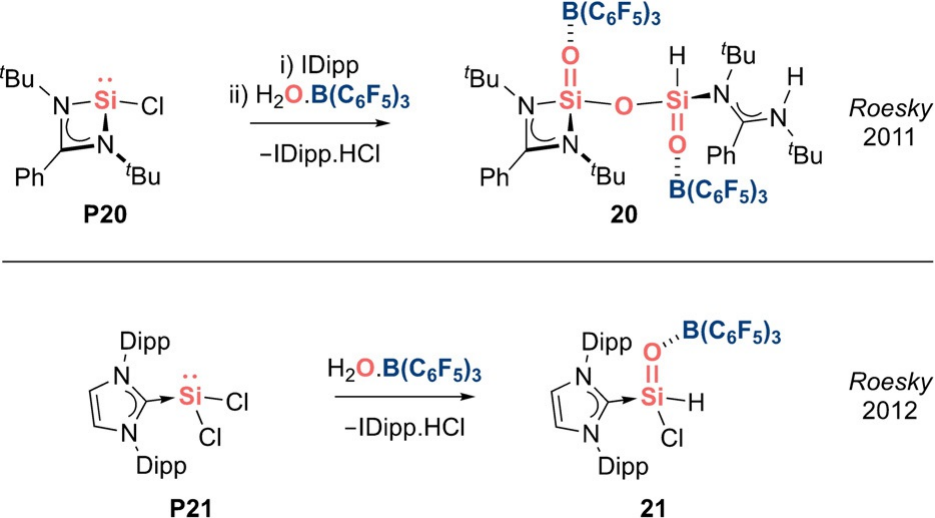
Roesky's acid–base stabilized silacarbonyls (Type **I**).

In 2011, Roesky et al. reported a sila‐acid anhydride **20** featuring a central O=Si−O−Si=O linkage with Si=O units stabilized by donor–acceptor interactions (Scheme 12, top).^[32a]^ In 2012, the same group employed a similar strategy to coordinatively trap silaformyl chloride **21** (Scheme 12, bottom).^[32b]^ Formyl chloride is an organic building block with great synthetic value, however its use is limited by its unstable nature, as it readily decomposes to CO and HCl at room temperature. Hence, this bottleable silaformyl chloride derivative can be anticipated to be a useful reagent to introduce the HSi=O functional group.

**Scheme 13:**
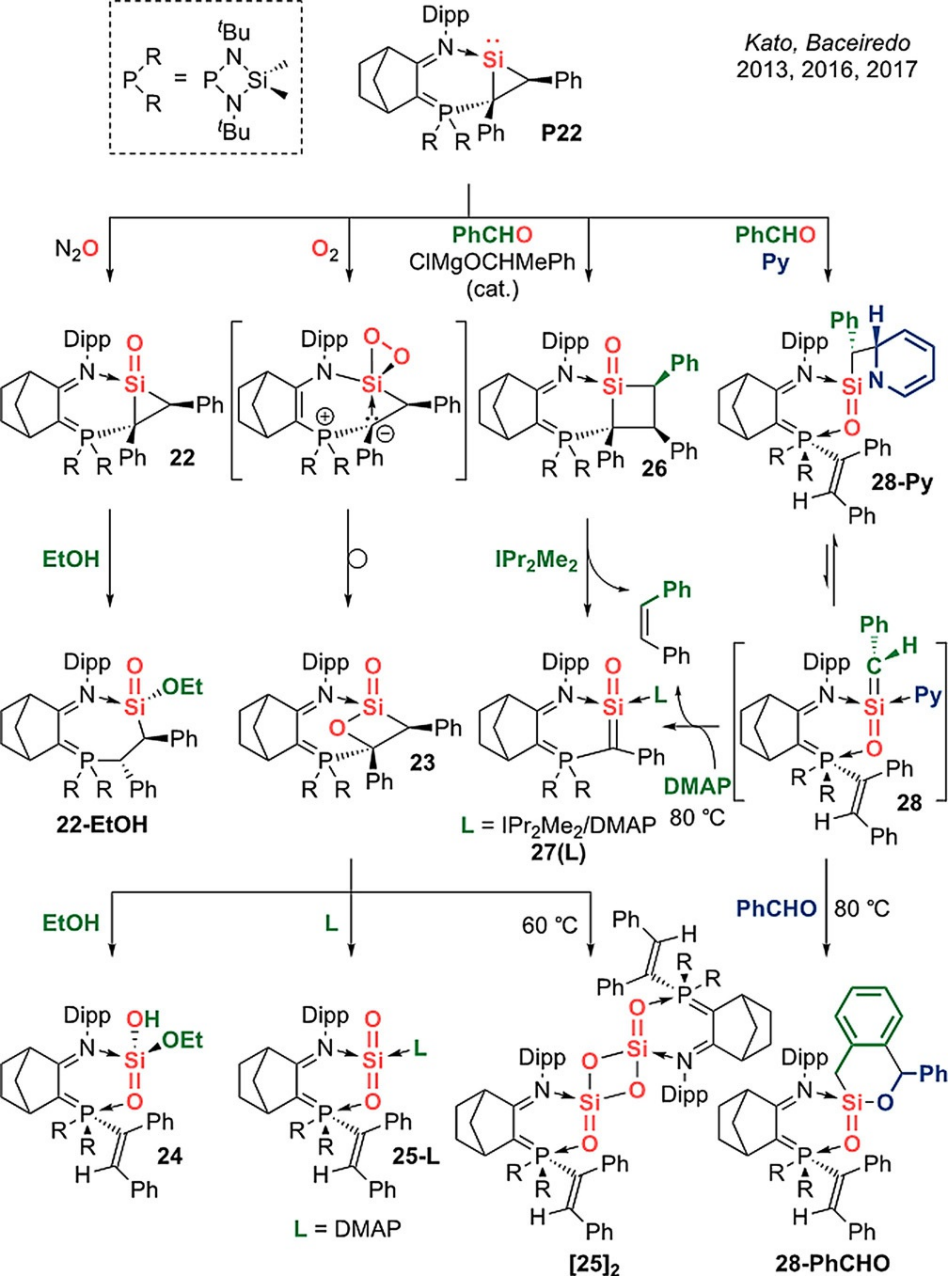
Kato and Baceiredo's acid–base stabilized silacarbonyls (Type **I**).

In 2013, Kato, Baceiredo et al. further elaborated the scope of acid–base stabilized silacarbonyl derivatives by employing an ambiphilic iminophosphorane supporting ligand (Scheme 13). Mono‐oxygenation of base‐stabilized silacycloprop‐1‐ylidene **P22** with N_2_O allows access to a base‐stabilized silacyclopropanan‐1‐one **22**, which represents a silicon analogue of the smallest cyclic ketone, which can be converted to base‐stabilized silaester **22‐EtOH** with ethanol.^[33a]^ Di‐oxygenation affords a base‐stabilized sila‐β‐lactone **23** via a pentacoordinate dioxasilirane intermediate which ensures the regio‐ and diastereospecific insertion of oxygen into the ylidic C−Si bond.^[33b]^ **23** can be further converted to acid–base stabilized silanoic acid **24** with ethanol, or generate acid–base stabilized SiO_2_ monomer **25‐DMAP** and its dimer **[25]_2_**.^[33c]^ Most remarkably, a Lewis acid catalyzes the reaction between silacycloprop‐1‐ylidene **P22** and benzaldehyde to afford a base‐stabilized silacyclobutanone **26**.^[33d]^ Further [2+2] cyclo‐reversion in the presence of IPr_2_Me_2_ releases *cis*‐stilbene to generate a base‐stabilized 1‐silaketene **27(IPr_2_Me_2_)** featuring cumulated C=Si=O double bonds.^[33d]^ Moreover, in the reaction of **P22** with benzaldehyde, performing the reaction in pyridine in the absence of a Lewis acid catalyst results in the transient formation of an acid–base stabilized 1‐silaketene intermediate **28**, which undergoes an unusual [2+2] cycloaddition with pyridine to afford sila‐β‐lactam **28‐Py**. Most notably, this cycloaddition is reversible at 80 °C to unveil the highly reactive intermediate **28** which can undergo a [4+2] cycloaddition/rearomatization sequence with benzaldehyde to form **28‐PhCHO**, or alternatively, in the presence of DMAP effect an intramolecular olefin metathesis to furnish *cis*‐stilbene and form base‐stabilized 1‐silaketene **27(DMAP)**, alluding to transition metal‐like behaviour at the silicon centre.^[33e]^

**Scheme 14:**
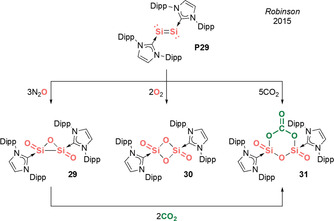
Robinson's NHC‐stabilized silicon oxides (Type **I**).

In 2015, Robinson et al. reported the isolation of remarkable NHC‐trapped molecular silicon oxides Si_2_O_3_ (**29**) and Si_2_O_4_ (**30**),[^34a^, ^34b^] and a mixed silicon/carbon oxide Si_2_CO_6_ (**31**)^[34c]^ via the controlled oxygenation of NHC‐stabilized disilicon **P29** with N_2_O, O_2_ or CO_2_, respectively (Scheme 14). Hence, NHC‐stabilized disilicon **P29** presents a unique molecular platform to mimic silicon surfaces and examine their oxidation to silicon oxides—a process that is highly relevant for the semiconductor and aviation industries.

**Scheme 15:**
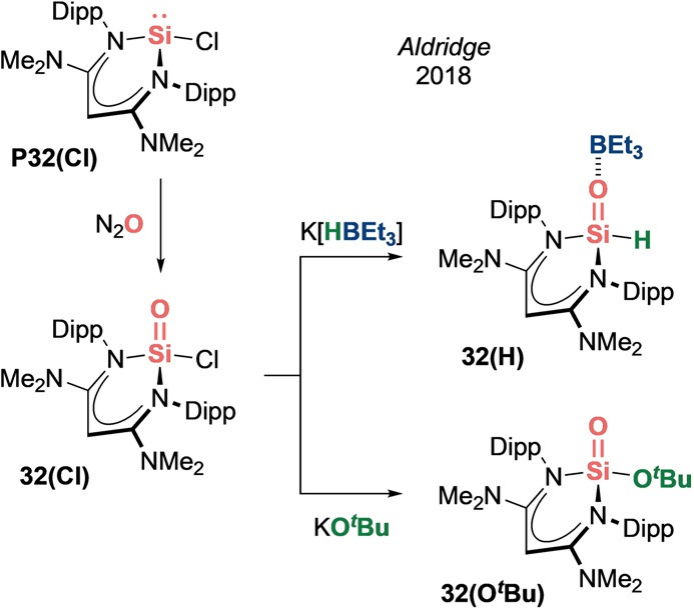
Aldridge's acid–base stabilized silacarbonyls (Type **I**).

In 2018, Aldridge et al. reported a base‐stabilized sila‐acyl chloride **32(Cl)** via direct oxygenation of a diamino functionalized β‐diketiminate‐supported chlorosilylene **P32(Cl)** (Scheme 15).^[35]^ **32(Cl)** proved to be a versatile building block that undergoes systematic carbonyl‐like reactions to access a sila‐aldehyde **32(H)** and a silaester **32(O** ^***t***^ **Bu)**.

**Scheme 16:**
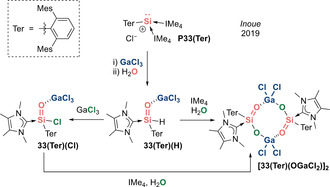
Inoue's acid–base stabilized silacarbonyls (Type **I**).

In 2019, Inoue et al. reported that an NHC‐stabilized silyliumylidene **P33(Ter)** undergoes hydrolysis in the presence of GaCl_3_ to afford acid–base stabilized sila‐aldehyde **33(Ter)(H)** (Scheme 16).^[36]^ Further hydrolysis of **33(Ter)(H)** resulted in the formation of a silacarboxylate dimer **[33(Ter)(OGaCl_2_)]_2_**. **33(Ter)(H)** also displays interesting H‐for‐Cl metathesis to yield acid–base stabilized sila‐acyl chloride **33(Ter)(Cl)**, which is a reversal of the typical Cl‐for‐H conversion, exemplified by the transformation from **32(Cl)** to **32(H)** (Scheme 15).

**Scheme 17:**
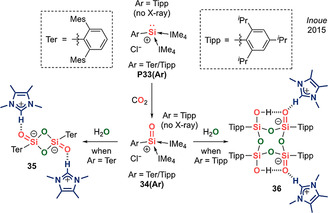
Inoue's base‐stabilized sila‐acylium ions **34(Ar)** (Type **II**) and their reactivities.

**A cationic analogue: [R(L)Si=O]^+^ (Type II)**. In 2015, Inoue et al. reported remarkable NHC‐stabilized sila‐acylium ions **34(Ar)** (Ar=Ter/Tipp) via oxygenation of NHC‐stabilized silyliumylidenes **P33(Ar)** with CO_2_ (Scheme 17).^[37]^ Hydrolysis of **34(Ter)** furnishes a dimeric silacarboxylate anion **35**, while the less bulky Tipp‐substituted **34(Tipp)** forms a cyclotetrasiloxanediol dianion **36**. These anionic silacarbonyl moieties are stabilized in the solid‐state by hydrogen bonds with imidazolium proton and/or neighbouring hydroxy proton.

**Scheme 18:**
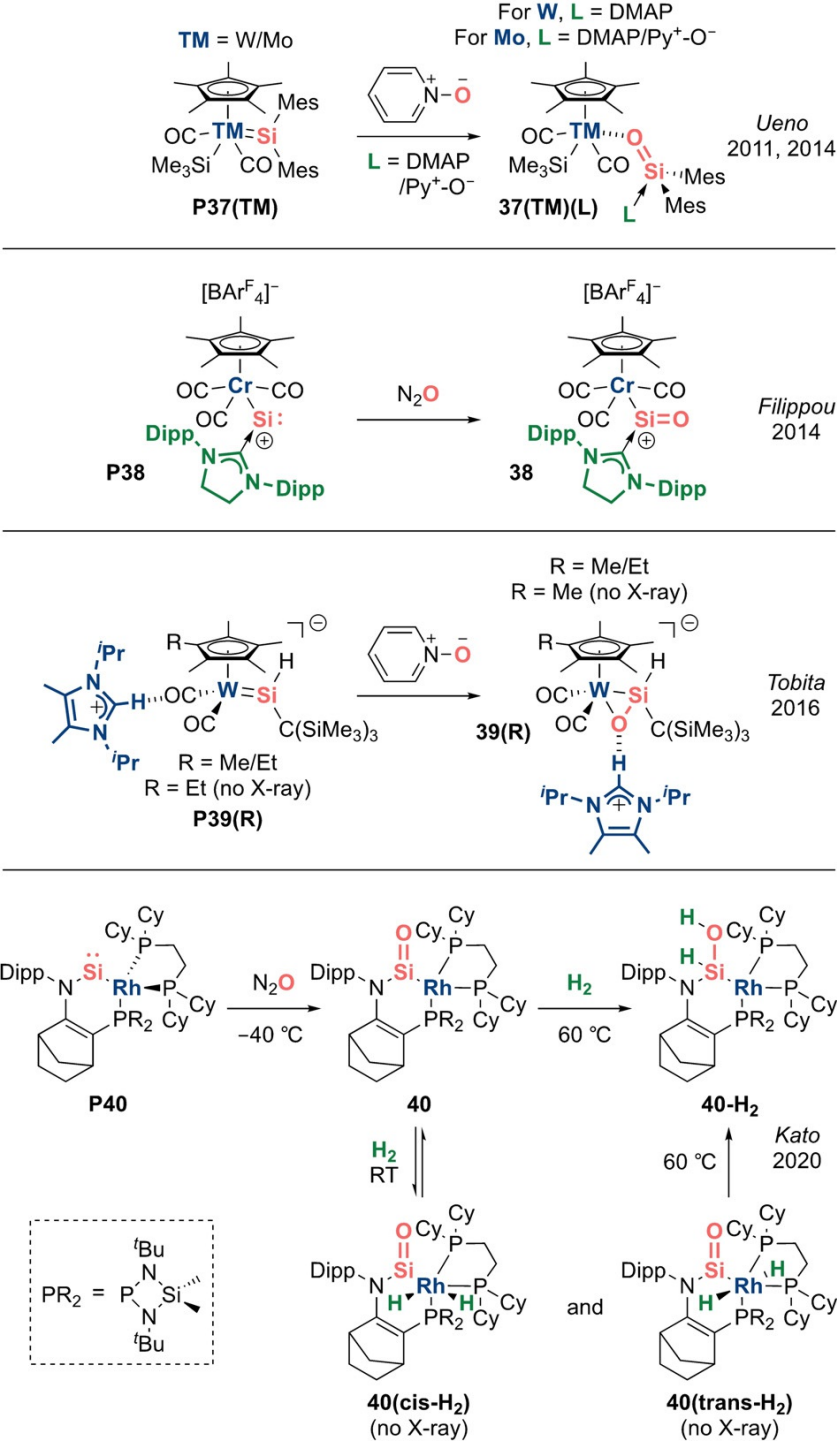
Transition metal‐stabilized silacarbonyls.

**Transition metal‐stabilized silacarbonyls**. In 2011, Ueno et al. reported complex **37(W)(DMAP)** featuring a silanone with η^1^ coordination to tungsten via the oxygen atom, while the silicon atom is stabilized via donation from DMAP (Scheme 18).^[38a]^ In 2014, the authors expanded the scope to include base‐stabilized silanone molybdenum complexes **37(Mo)(L)** (L=DMAP/Py^+^‐O^−^).^[38b]^

In 2014, Filippou et al. reported an unusual reverse coordination of the Si=O fragment in which silicon is bound in an η^1^ fashion to the transition metal.[^38c^, ^38d^] Complex **38** contains a trigonal planar silicon centre with an exceptionally short double bond with the terminal oxygen atom (1.523(3) Å), and can be regarded as either an sila‐acyl chromium complex or NHC‐stabilized silicon monoxide complex (Scheme 18).

In 2016, Tobita et al. reported η^2^ coordination of a sila‐aldehyde to tungsten in complex **39(R)** (R=Me/Et) (Scheme 18).^[38e]^ Strong π back‐donation from the anionic transition metal fragment results in metallacycle formation instead of a π complex.

In 2020, Kato et al. described a sila‐acyl rhodium complex **40** featuring a three‐coordinate silicon centre with short Si=O double bond of 1.540(3) Å (Scheme 18).^[38f]^ Interestingly, while **40** undergoes reversible uptake of H_2_ at the Rh^I^ centre at room temperature, hydrogenation of the sila‐acyl moiety can be achieved at 60 °C to form **40‐H_2_**. DFT analysis revealed hydrogen transfer to occur via a series of H‐migrations from rhodium to the Si=O fragment, reminiscent of the mechanism of the Fischer–Tropsch process.^[39]^ It should be noted that direct hydrogenation of a silacarbonyl with H_2_ has not been reported to date, and Aldridge and Goicoechea's alumacarbonyl **[15‐THF]_2_** remains the only example of a main group carbonyl analogue to showcase such FLP‐type reactivity. Hence, this work hints at future opportunities for cooperative bond activation by pairing transition metals with main group carbonyls.

### 3.3 Acid–Base Free Silacarbonyls: “Kipping's Dream”

**Figure 2:**
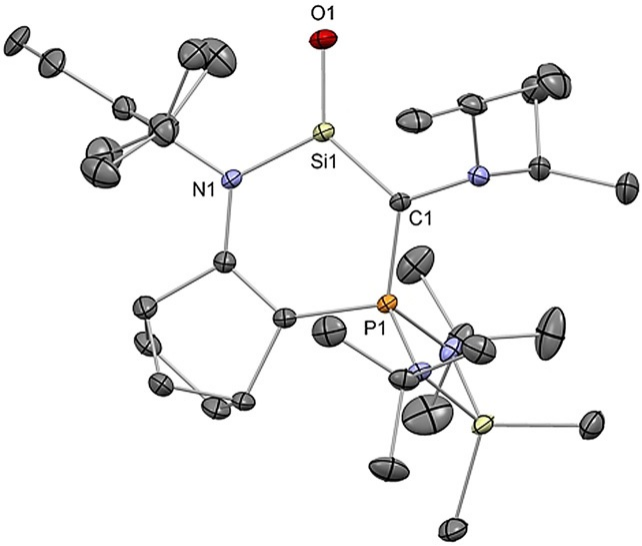
Solid‐state structure of **41(** ^***i***^ **Pr)**. For clarity, hydrogen atoms are omitted. Thermal ellipsoids set at 50 % probability.

**Scheme 19:**
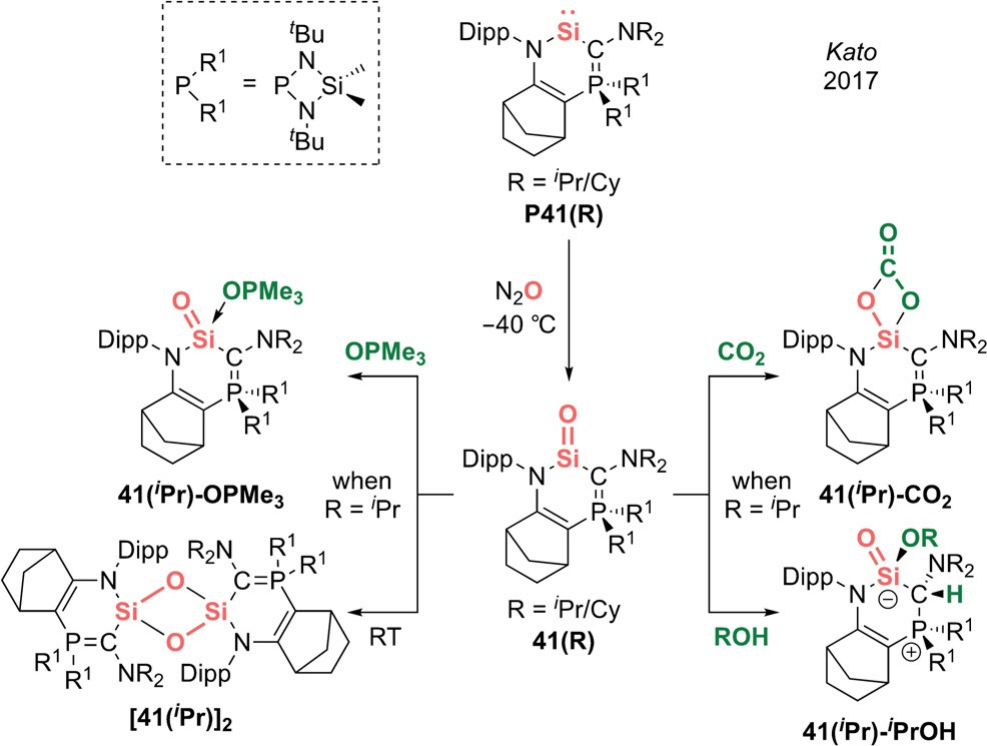
Kato's acid–base free cyclic (amino)(ylide)silacarbonyl **41(R)** (Type **I**) and its reactivity.

In 2017, Kato et al. reported the landmark isolation of acid–base free silanones (Type **I**) in crystalline form, thereby representing the fulfilment of “Kipping's dream”.^[8]^ Silacarbonyls **41(R)** (R=^*i*^Pr/Cy) were generated via oxygenation of electron‐rich cyclic (amino)(ylide)silylenes **P41(R)** at −40 °C (Scheme 19). While **41(** ^***i***^ **Pr)** dimerizes to cyclodisiloxane **[41(** ^***i***^ **Pr)]_2_** at room temperature (with a half‐life of 0.5 h), NCy_2_ substitution in **41(Cy)** significantly enhances its persistence at room temperature (half‐life: 5 h), suggesting the key influence of steric factors in the stability of these systems. ^29^Si NMR shows a signal at 38.4 ppm for **41(** ^***i***^ **Pr)**, which is downfield‐shifted as compared to donor‐stabilized silacarbonyl species (−55 to −91 ppm). X‐ray diffraction analysis unambiguously revealed **41(** ^***i***^ **Pr)** to feature a trigonal planar geometry around silicon with a terminal oxygen atom (Figure 2). The Si=O double bond length of 1.533(1) Å is at the shorter end of the range reported for base‐stabilized silacarbonyls (1.531–1.579 Å). The Si−C bond length (1.773(2) Å) approaches that for the polarized Si=C double bond of a Brook‐type silene (1.764 Å), while the Si−N bond (1.731(2) Å) is similar to Driess’ donor‐stabilized silaureas (**17‐L** and **17‐O=L**: 1.732(2)–1.754(3) Å), signifying that internal π donation from the ylide obviates the need for external bases to stabilize the electron deficient Si=O fragment. DFT analysis revealed a WBI of 1.14 for the Si=O double bond in **41(** ^***i***^ **Pr)** and significant charge polarization between Si (+2.16) and O (−1.24).

With a uniquely unperturbed sila‐amide in hand, the authors explored its reactivity with various small molecules. Its latent Lewis acidity was confirmed via adduct formation with OPMe_3_ and [2+2] cycloaddition with CO_2_ to afford **41(** ^***i***^ **Pr)‐OPMe_3_** and **41(** ^***i***^ **Pr)‐CO_2_**, respectively (Scheme 19). Treating **41(** ^***i***^ **Pr)** with ^*i*^PrOH results in the formation of a set of keto‐enol‐like tautomers in which the proton undergoes rapid 1,3‐migration between oxygen and the ylidic carbon. While this equilibrium strongly favours the silanol form in solution, a base‐stabilized silacarbamate **41(** ^***i***^ **Pr)‐** ^***i***^ **PrOH** was obtained in the solid state.

**Figure 3:**
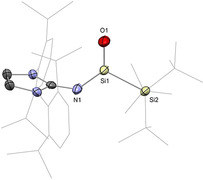
Solid‐state structure of **42(** ^***t***^ **Bu)**. For clarity, hydrogen atoms are omitted, Dipp and ^*t*^Bu groups are simplified as wireframe. Thermal ellipsoids set at 50 % probability.

**Scheme 20:**
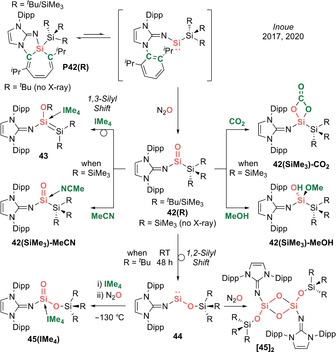
Inoue's acid–base free acyclic (imino)(silyl)silacarbonyl **42(R)** (Type **I**) and its reactivity.

Three months later, in 2017, Inoue et al. described the remarkable isolation of crystalline acyclic silanones (Type **I**).^[9a]^ While the precursor (imino)(silyl)silylenes are highly reactive species and “mask” themselves by inserting into aromatic C=C double bonds to form silepins **P42(R)** (R=^*t*^Bu/SiMe_3_), the silylene form could be “unmasked” in the presence of N_2_O to afford the acid–base free acyclic (imino)(silyl)silacarbonyls **42(R)** (Scheme 20). These systems are remarkably stable, with room temperature half‐lives of 7 h for **42(SiMe_3_)** and 24 h for **42(** ^***t***^ **Bu)** in solution, and are indefinitely stable in the solid state at −30 °C (for **42(SiMe_3_)**) and at room temperature (for **42(** ^***t***^ **Bu)**). ^29^Si NMR signals at 33.7 ppm for **42(SiMe_3_)** and 28.8 ppm for **42(** ^***t***^ **Bu)** are similar to Kato's N,C‐silacarbonyl **41(** ^***i***^ **Pr)** (38.4 ppm). X‐ray diffraction analysis confirmed the monomeric nature of **42(** ^***t***^ **Bu)** with a three‐coordinate silicon centre and terminal oxygen atom (Figure 3). The Si=O double bond length of 1.537(3) Å is similar to **41(** ^***i***^ **Pr)** (1.533(1) Å). The shortened Si−N bond (1.646(3) Å) and elongated exocyclic C=N double bond suggest the strong influence of the NHI substituent, offering ylidic stabilization of the Si=O fragment. This was further confirmed via DFT analysis of **42(** ^***t***^ **Bu)**, in which the HOMO−10 represents the Si=O π bond, with a pronounced contribution from the exocyclic nitrogen of the NHI ligand. WBI gives a value of 1.13 for the Si=O bond, similar with **41(** ^***i***^ **Pr)** (1.14), which seems to suggest a similar degree of perturbation by the π donor substituents. Notably, the positive charge on Si (+1.70) is drastically reduced as compared with **41(** ^***i***^ **Pr)** (+2.16), which can be attributed to the strong σ donating abilities of the silyl ligand. Overall, the NHI–silyl ligand pair, featuring the complementary action of NHI as a strong π donor offering outer‐sphere protection and the silyl ligand as a strong σ donor offering steric bulk in the immediate vicinity of the Si=O motif, seems tailor‐made to tame acyclic silacarbonyls.

Investigation of the reactivity of silacarbonyl **42(SiMe_3_)** with small molecules has also taken place: uptake of CO_2_ occurs via [2+2] cycloaddition to form **42(SiMe_3_)‐CO_2_**; 1,2‐addition of MeOH to form the silanol **42(SiMe_3_)‐MeOH** is also reported (Scheme 20). It is interesting to note that the silanol is formed exclusively, despite the possibility for protonation of the ylidic nitrogen, unlike Kato's **41(** ^***i***^ **Pr)‐** ^***i***^ **PrOH**, in which the ylidic carbon has been protonated. The authors also investigated the decomposition pathways of acyclic silanone systems. Silacarbonyl **42(SiMe_3_)** decomposes in C_6_D_6_ within 14 h to a series of unidentified products, possibly via 1,3‐silyl migration of a SiMe_3_ group from the Si(SiMe_3_)_3_ ligand to the terminal oxygen to give an intermediate disilene, which undergoes further decomposition via activation of the NHI ligand. However, in the presence of weakly basic MeCN, a base‐stabilized silanone **42(SiMe_3_)‐MeCN** could be isolated and structurally characterized. When stronger bases such as IMe_4_ or THF were employed, the proposed disilenes were immediately formed as their base adducts, and in the case of the more basic IMe_4_, disilene **43** was sufficiently stable to permit structural authentication. On the other hand, silacarbonyl **42(** ^***t***^ **Bu)** undergoes a remarkable Brook‐type 1,2‐silyl migration of the Si^*t*^Bu_3_ ligand to the terminal oxygen to furnish an acyclic dicoordinate (imino)(siloxy)silylene **44**. These silyl migrations, driven by the highly oxophilic nature of silicon coupled with its strong desire to form new Si−O single bonds (rather than Si=O double bonds), remarkably overcome the counter‐intuitive oxidation state changes from Si^IV^ to Si^II^. Furthermore, this N,O‐silylene **44** has recently been shown to be able to undergo oxidation to furnish a transient N,O‐silacarbonyl which can be trapped by an NHC to furnish **45(IMe_4_)**.^[9b]^ However, it dimerizes in the absence of an external base, highlighting the integral role of the silyl ligand to stabilize the Si=O moiety. The overall transformation from silepin **P42(** ^***t***^ **Bu)** to N,O‐silacarbonyl **45(IMe_4_)** involves multiple oxidation state changes of the central silicon atom, i.e. Si^IV^–Si^II^–Si^IV^–Si^II^–Si^IV^, hinting at the versatility of such NHI‐supported silicon species for possible future catalytic processes involving silicon centres.

**Scheme 21:**
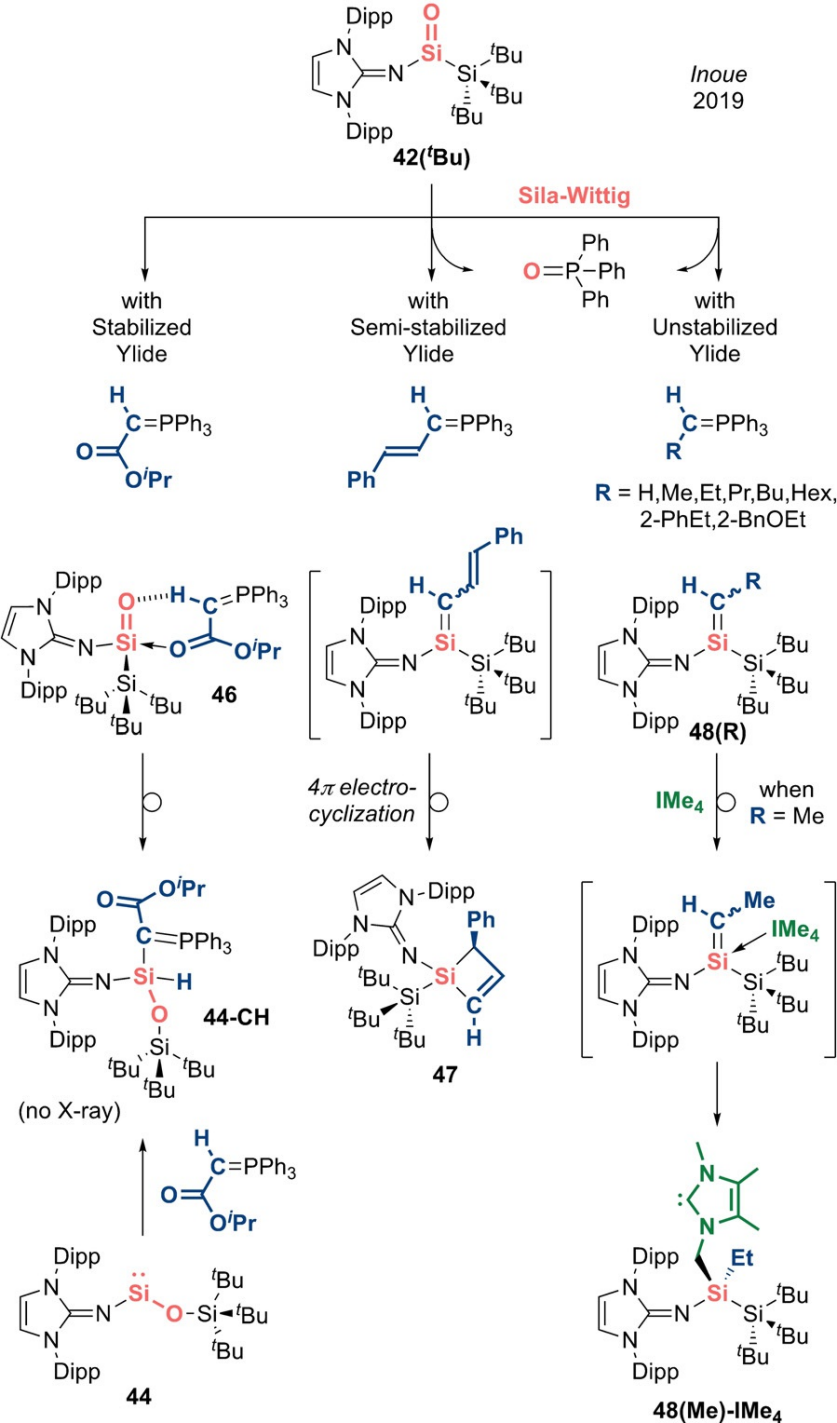
Sila‐Wittig chemistry with acid–base free silacarbonyl **42(** ^***t***^ **Bu)**.

The Wittig reaction in which a carbonyl compound is converted to an alkene by a phosphorus ylide is a powerful synthetic tool in the toolbox of organic chemists which surpasses all other olefination methods. More recently, Inoue et al. reported a sila‐Wittig reaction in which a silacarbonyl can undergo heavier olefination with phosphorus ylides to generate a series of silenes, elegantly mimicking the classical Wittig reaction.^[9c]^ The nature of the ylide plays an integral role to determine the selectivity of products (i.e. (*E*)/(*Z*)‐alkenes based on thermodynamic/kinetic control). Hence, the authors investigated the reactivity of acid–base free N,Si‐silacarbonyl **42(** ^***t***^ **Bu)** with stabilized, semi‐stabilized and unstabilized ylides (Scheme 21). With the stabilized ylide Ph_3_P=C(H)COO(^*i*^Pr), no sila‐Wittig reaction resulted, as evidenced from the lack of O=PPh_3_ formation. Instead, the silacarbonyl–ylide adduct **46** was formed, in which the ylide acts as an amphiphilic electron donor and acceptor simultaneously. This adduct is thermally unstable and collapses to a Si−H containing species, as verified by ^1^H NMR. The authors further posit its formation as involving initial dissociation of the ylide followed by rearrangement of silacarbonyl **42(** ^***t***^ **Bu)** to N,O‐silylene **44**, which is able to activate the ylidic C−H bond of the phosphorus ylide. This assumption is further supported by the fact that **44‐CH** can be acquired directly by treating **44** with the free ylide.

With semi‐stabilized ylide (*E*)‐Ph_3_P=C(H)C=C(H)Ph, the detection of O=PPh_3_ in the ^31^P NMR hints at successful Si=O‐to‐Si=C metathesis (Scheme 21). However, X‐ray diffraction analysis revealed not the expected silene product but a silacyclobutane **47**, presumably formed via 4π‐electrocyclization of the original silene. Motivated by this, the authors moved on to unstabilized ylides. A successful sila‐Wittig reaction was indicated by the formation of O=PPh_3_ in the ^31^P NMR spectrum, affording a plethora of silenes **48(R)** (R=H, Me, Et, Pr, Bu, Hex, 2‐PhEt, 2‐BnOEt) themselves evidenced by their ^29^Si NMR shifts (42.6–56.3 ppm). NOESY experiments revealed high *Z*‐selectivity, in analogy with the classical Wittig reaction, which tends to be highly selective for (*Z*)‐alkenes when unstabilized ylides are employed. However, in contrast, the removal of O=PPh_3_ is impossible as the highly reactive (imino)(silyl)silene products **48(R)** decompose upon work‐up. Hence, the authors attempted to isolate these transient silenes as their NHC adducts. Employing IMe_4_ resulted in intramolecular activation of the wingtip C(sp^3^)−H bond of the NHC across the Si=C double bond to form a silyl‐substituted NHC **48(Me)‐IMe_4_**, which was identified by X‐ray diffraction. The observed instability of the silene–NHC adduct is in line with the highly reactive nature of (imino)(silyl)silenes. This work represents proof of concept that unperturbed a heavier silacarbonyl can not only exhibit carbonyl‐like reactivity, but also mimic the activity of transition metals by acting as a platform for oxide ion transfer chemistry, as exemplified by the sila‐Wittig reaction.

**Figure 4:**
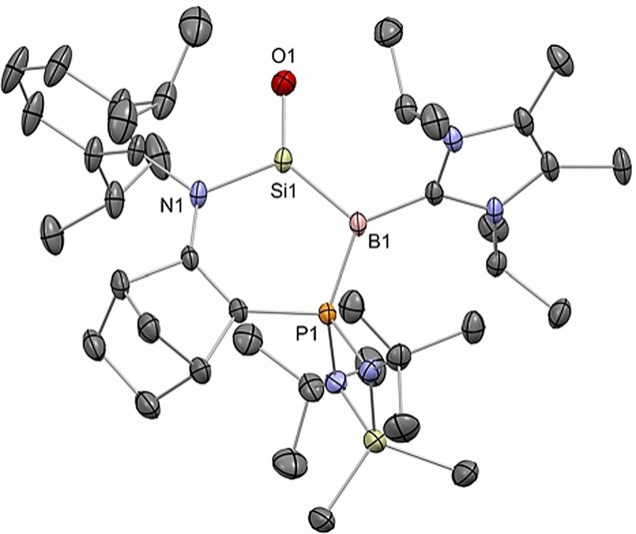
Solid‐state structure of **49**. For clarity, hydrogen atoms are omitted. Thermal ellipsoids set at 50 % probability.

**Scheme 22:**
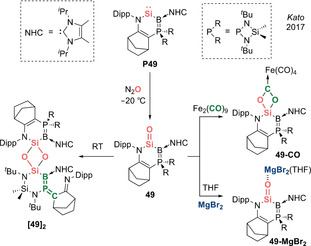
Kato's cyclic (amino)(bora‐ylide)silacarbonyl **49** (Type **I**) and its reactivity.

In 2017, simultaneous with Inoue's report on acyclic silanones, Kato et al. described the isolation of a crystalline cyclic (amino)(bora‐ylide)silanone **49** (Type **I**) via oxygenation of a cyclic N,B‐silylene **P49** with N_2_O (Scheme 22).^[10]^ Substituting the carbo‐ylide functionality with an exceptionally strong π donating bora‐ylide leads to a dramatic increase in the half‐life from 0.5 h for N,C‐silacarbonyl **41(** ^***i***^ **Pr)** to 4 days for N,B‐silacarbonyl **49**. The ^29^Si NMR spectrum of the latter shows a broad quartet at 71.3 ppm, which is slightly downshifted compared with **41(** ^***i***^ **Pr)** (38.4 ppm) and Inoue's N,Si‐silacarbonyl **42(** ^***t***^ **Bu)** (28.8 ppm). X‐ray diffraction analysis confirms the tricoordinate nature of both the central silicon and the adjacent boron atom (Figure 4). The Si=O bond (1.5432(12) Å) is slightly elongated as compared with **41(** ^***i***^ **Pr)** (1.533(1) Å) and **42(** ^***t***^ **Bu)** (1.537(3) Å). The B−Si bond (1.899(2) Å) is rather short, approaching those of borasilenes containing B=Si double bonds (1.838–1.859 Å) while the N−Si bond (1.763(2) Å) is significantly elongated as compared with **41(** ^***i***^ **Pr)** (1.731(2) Å). Hence, the unique stability of N,B‐silacarbonyl **49** can be ascribed to the enhanced perturbation of the Si=O fragment by an ylidic ligand based on the more electropositive boron. DFT analysis further confirms this assessment, as the Gibbs free energy for dimerization of **49** (−67 kJ mol^−1^) is approximately half that of **41(** ^***i***^ **Pr)** (−126 kJ mol^−1^). The WBI of 1.09 for the Si=O moiety is reduced compared to other tricoordinate silacarbonyls **41(** ^***i***^ **Pr)** (1.14) and **42(** ^***t***^ **Bu)** (1.13), further verifying the strong electronic perturbation of the Si=O fragment. While the HOMO is centred on the ylidic boron ligand, the Si=O π and π^*^ orbitals can be located in the HOMO−8 and LUMO+6, respectively.

In spite of extensive electronic and kinetic stabilization, dimerization of N,B‐silacarbonyl **49** still occurs at room temperature (albeit slowly; Scheme 22). However, X‐ray diffraction analysis of **[49]_2_** revealed no simple cyclodisiloxane product. The authors postulated that initial head‐to‐tail dimerization forms a highly strained cyclodisiloxane, and the relief of steric congestion within the four‐membered ring drives the onward ligand rearrangement. Interestingly, this dimer features a (borylene)(methylene)phosphorane function with cumulative B=P=C double bonds and is the first example of such which incorporates a group 13 element. The stability of **49** could be further increased by capping the terminal oxygen with a Lewis acid such as MgBr_2_, and **49‐MgBr_2_** is stable in solution for more than three weeks. The solid‐state structure revealed only slight lengthening of the Si=O bond to 1.553(2) Å (0.6 %), while both the B−Si (1.865(3) Å) and N−Si (1.744(2) Å) bonds experienced substantial shortening. Furthermore, **49‐MgBr_2_** represents the first example of a base‐free silacarbonyl coordinated only by a Lewis acid. This suggests that **49** has a unique nucleophilic character, as opposed to the more typical electrophilic nature associated with such systems. **49** can also undergo a [2+2] cycloaddition with small molecules such as CO (employing Fe_2_(CO)_9_ as the CO source) to form a four‐membered cyclic dioxocarbene Fe(CO)_4_ complex **49‐CO**.

**Figure 5:**
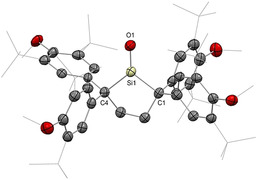
Solid‐state structure of **50**. For clarity, hydrogen atoms are omitted, Me and ^*t*^Bu groups are simplified as wireframe. Thermal ellipsoids set at 50 % probability.

**Scheme 23:**
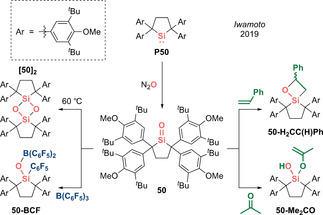
Iwamoto's cyclic di(alkyl)silanone **50** (Type **I**) and its reactivity.

While the incorporation of exceptionally strong internal π donor functionalities (i.e. amino, ylide, NHI, bora‐ylide) has replaced the need for external bases to tame base‐free silacarbonyls, inevitable electronic perturbation of the of the central Si=O functionality distinguishes these silacarbonyls from silanones, which might be regarded as the true homologues of ketone. “Genuine” silanones have remained elusive for more than 100 years, until recently when Iwamoto et al. described the breakthrough synthesis/isolation of a crystalline cyclic di(alkyl)silanone **50** (Type **I**) which is stable at room temperature.^[13]^ This significant feat was achieved through an arduous six‐step synthesis to the precursor silylene **P50**, followed by final oxygenation with N_2_O (Scheme 23). The ^29^Si NMR spectrum for **50** displays a resonance at 90.0 ppm that is downfield‐shifted compared with other silacarbonyls featuring strong π donor substituents (**41(** ^***i***^ **Pr)**, **42(** ^***t***^ **Bu)**, **49**: 28.8–71.3 ppm). X‐ray diffraction analysis unequivocally confirms the planar three‐coordinate nature of the silanone **50** with a Si=O bond length of 1.518(2) Å, which is noticeably shorter than other acid–base free silacarbonyls (**41(** ^***i***^ **Pr)**, **42(** ^***t***^ **Bu)**, **49**: 1.533(1)–1.5432(12) Å) and very close to H_2_Si=O (1.515 Å) determined by rotational spectroscopy, hence hinting at the unperturbed nature of the Si=O moiety (Figure 5). This hypothesis was affirmed by DFT analysis, which revealed the WBI of the Si=O function to be 1.35, that is, significantly greater than other silacarbonyl systems (**41(** ^***i***^ **Pr)**, **42(** ^***t***^ **Bu)**, **49**: 1.09–1.14). The Si=O π and π^*^ orbitals are located in the HOMO−13 and LUMO, respectively. While the charge on the central silicon atom is strongly influenced by the σ donating qualities of the adjacent substituents, the charge on the terminal oxygen is determined by the π donor properties, thus making it a good measure for the π donating capacity of its substituents. For silanone **50** (Si: +2.08, O: −1.10), the lack of π donors substantially reduces the charge on oxygen as compared with strong π donor substituted silacarbonyls (**41(** ^***i***^ **Pr)**, **42(** ^***t***^ **Bu)**, **49**: −1.23 to −1.27), hence supporting the notion that its stability is purely based on kinetic protection. While this huge steric profile underpins its remarkable stability in solution at room temperature, it is insufficient to completely inhibit head‐to‐tail dimerization, which is still possible at 60 °C to afford a very congested cyclodisiloxane **[50]_2_**.

With a “genuine” Si=O motif in hand, the authors began to explore its reactivity. In contrast to Kato's N,B‐silacarbonyl **49**, which prolongs its half‐life by forming stable adducts with Lewis acids, the adduct of silanone **50** and B(C_6_F_5_)_3_ collapses via 1,3‐C_6_F_5_ migration from boron to silicon, in a similar fashion to Roesky's alumacarbonyl **13** and in line with the electrophilic character of **50** (Scheme 23). While an ene reaction with the polarized C=O double bond of acetone is possible, [2+2] cycloaddition with the weakly polarized C=C double bond of styrene to form **50‐H_2_CC(H)Ph** confirms the strong ambiphilicity intrinsic in the Si=O motif. This work affirms the integral role of kinetic stabilization to tame acid–base free silanones, giving rise to a spectrum of isolable silacarbonyl derivatives with a variety of substitution patterns. Hence, this opens avenues for comparison with their lighter homologues, advancing the vision of Kipping and bringing his dream to reality.

### 3.4 Organogermanium Oxides

Germacarbonyls containing Ge=O double bonds are even rarer than their silicon counterparts. Although the first evidence of these germanium homologues of ketones was reported by Satgé et al. in 1971,^[40]^ it was not until 2009 when Driess et al. isolated crystalline germanones by taking advantage of stabilization provided by additional bases. In 2012, the seminal discovery by Tamao, Matsuo et al. of a purely kinetically stabilized “genuine” germanone represents the first heavier carbonyl among any of the group 14 elements. While this motivated many recent breakthroughs, this germanone maintains its position as the heaviest carbonyl analogue reported to date.

### 3.5 Acid–Base Stabilized Germacarbonyls

**Scheme 24:**
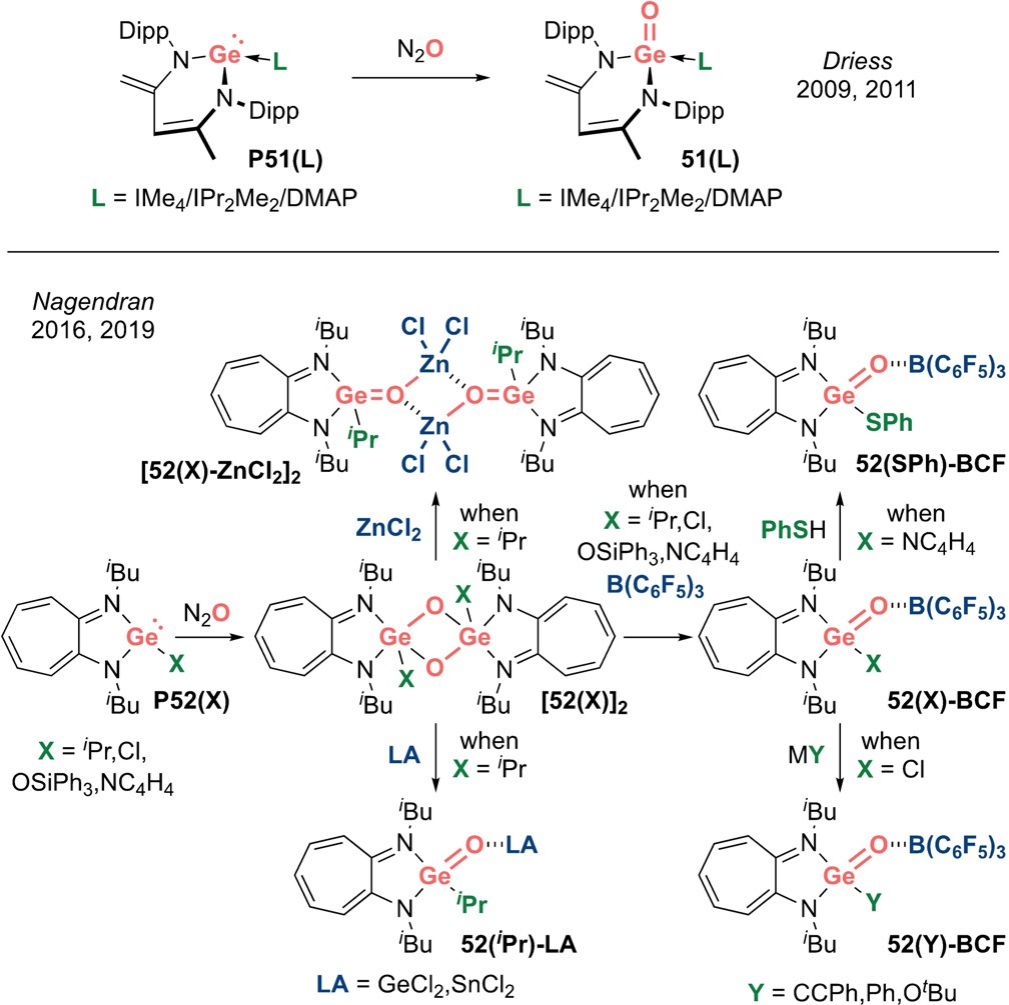
Acid–base stabilized germacarbonyls (Type **I**).

**Neutral analogues: R_2_Ge=O (Type I)**. In 2009, Driess et al. reported the first examples of isolable base‐stabilized germacarbonyls **51(L)** (L=IMe_4_/IPr_2_Me_2_) (Scheme 24, top).^[41a]^ The substantial increase in nucleophilicity of the N‐heterocyclic germylene unit brought about by the coordination of a strong donor NHC ligand leads to ready oxidation, even with N_2_O, in line with the similar trend observed for lighter silicon analogues. In 2011, Driess et al. further reported that when a weaker donor such as DMAP was employed, germacarbonyl **51(DMAP)** features a significantly shortened Ge=O bond (1.646(2) Å) as compared with the **51(IMe_4_)** (1.672(3) Å) and **51(IPr_2_Me_2_)** (1.664(2) and 1.670(2) Å), in line with the smaller degree of perturbation caused by the DMAP donor.^[41b]^

In 2016, Nagendran et al. reported an N_2_O oxygenation of an aminotroponiminate‐supported germylene **P52(** ^***i***^ **Pr)** to furnish a cyclodigermoxane **[52(** ^***i***^ **Pr)]_2_**, presumably via dimerization of a transient germanone (Scheme 24, bottom).^[41c]^ Treating the dimer with Lewis acids affords acid–base stabilized germanones **52(** ^***i***^ **Pr)‐LA** (LA=B(C_6_F_5_)_3_/GeCl_2_/SnCl_2_/ZnCl_2_). In 2019, the same group elaborated on this chemistry by varying the X substituent on the germylene **P52(X)** (X=^*i*^Pr, Cl, OSiPh_3_, NC_4_H_4_) to access a series of isolable Lewis acid–base stabilized germacarbonyls with significantly elongated Ge=O double bonds (1.695(3)–1.728(5) Å).^[41d]^

### 3.6 An Acid–Base Free Germanone: The First Heavy Carbonyl

**Figure 6:**
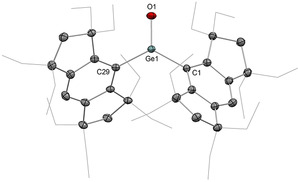
Solid‐state structure of **53**. For clarity, hydrogen atoms are omitted, Et groups are simplified as wireframe. Thermal ellipsoids set at 50 % probability.

**Scheme 25:**
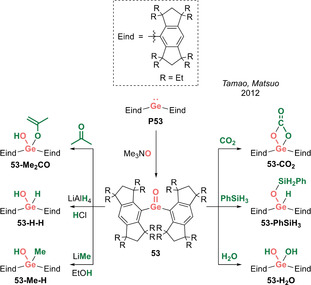
Tamao and Matsuo's acid–base free germanone **53** (Type **I**) and its reactivity.

In 2012, the landmark discovery of the first heavier carbonyl analogue by Tamao, Matsuo et al. was based on an acyclic acid–base free germanone (Type **I**).[^6a^, ^6b^] This remarkable achievement was enabled by the deployment of the extremely bulky and rigid Eind ligand scaffold (Scheme 25). Treating bis(Eind)germylene **P53** with Me_3_NO as the oxygen atom transfer reagent yielded bis(Eind)germanone **53**, which has remarkable thermal stability up to 200 °C. X‐ray diffraction analysis unambiguously revealed the planar tricoordinate nature of the central germanium atom affixed with a terminal oxygen atom (Figure 6). While the sheer size of the Eind ligand is apparent from the solid‐state structure, another key aspect is the rigidity imbued by its fused ring structure, restricting motion of the Eind ligands on either side of the Ge=O moiety, effectively obviating any potential side reactions (such as C−H activation) that have plagued previous attempted syntheses of such species. The Ge=O bond length of 1.6468(5) Å is at the shorter end of the range defined by base‐stabilized germaureas (**51(L)**: 1.646(2)–1.672(3) Å). DFT analysis revealed a WBI of 1.25 for the Ge=O fragment, with NPA charges of +1.80 (Ge) and −1.05 (O), indicating the important contribution of the ylidic germylene oxide (Eind)_2_Ge^+^−O^−^ form. The Ge=O π and π^*^ orbitals are represented in the HOMO−5 and LUMO, respectively, and the HOMO is composed of the non‐bonding oxygen lone pair. An in‐depth computational study by Pandey in 2015 highlighted the importance of non‐covalent London dispersion interactions provided by the Eind substituents towards the overall stability of **53**.^[6c]^

The same team subsequently probed the reactivity of this germanone, with a view to elucidating similarities and differences from lighter ketone homologues. While analogous reduction with LiAlH_4_ could be carried out, reduction by milder PhSiH_3_ proceeds without the need for a catalyst to afford **53‐H‐H** and **53‐PhSiH_3_**, respectively (Scheme 25). Familiar nucleophilic addition reactions with MeLi or H_2_O yielded the expected methylated germanol **53‐Me‐H** and hydroxylated digermanol **53‐H_2_O**, respectively. Most interestingly, significant differences are observed in reactivity towards C=O containing compounds. With acetone, an ene reaction occurs, which is uncommon with two carbonyl compounds. This presumably reflects the much higher basicity of the terminal oxygen atom in **53**. Lastly, the latent polarization within the Ge=O double bond facilitates binding of CO_2_ in a [2+2] fashion to form **53‐CO_2_**, a reaction that is energetically unfavourable for standard ketones. Perhaps most importantly, this work sparked excitement by offering the possibility of bottleable acid–base free main group carbonyl analogues, and it might be said that it paved the way for more recent syntheses of carbonyl analogues from across the Periodic Table.

## 4 Group 15 Carbonyl Analogues

Oxoammonium ions are the isoelectronic nitrogen analogues of carbonyl compounds. In particular, those derived from [TEMPO]^+^ are widely employed as catalytic oxidants in the dehydrogenation of alcohols to carbonyl compounds, due to the unique stability of the TEMPO radical.^[42a]^ However, it was not until 2007 that Nishide et al. reported the structural authentication of [TEMPO]^+^, representing the first isolation of a nitrogen analogue isoelectronic with carbonyl compounds (Type **I**).^[42b]^ This system features a trigonal planar nitrogen centre and a short N=O double bond (1.184(10) Å). On the other hand, the only isolable heavier group 15 carbonyl analogues reported so far are the phosphacarbonyls.

### 4.1 Organophosphorus Oxides

Phosphorus and carbon share a diagonal relationship in the Periodic Table, and organophosphorus compounds have been described as “carbon copies”. In stark contrast with other main group elements, organophosphorus oxides containing PO single bonds and double bonds are equally prevalent. In fact, the high oxophilicity of phosphorus and the latent P=O double bond strength (536 kJ mol^−1^) drive the Michaelis–Arbuzov reaction which transforms the P−O single bonds of phosphites to the P=O double bonds of phosphine oxides.^[43]^ The classical Wittig reaction also exploits the formation of a P=O double bond to drive conversion of the C=O double bonds of ketones to C=C double bonds of alkenes (which are less stable by ca. 126 kJ mol^−1^).^[43]^ The inherent stability of these tetrahedral σ^4^λ^5^‐phosphine oxides can be partly ascribed to steric protection of the electrophilic phosphorus centre. Hence, σ^3^λ^5^‐phosphacarbonyl cations featuring a trigonal planar phosphorus environment, and which are isoelectronic with carbonyl compounds, are elusive synthetic targets. Here, we survey the development of phosphacarbonyls from their initial isolation as base‐stabilized entities to the recent discovery of base‐free phosphacarbonyl cations.

### 4.2 Base‐Stabilized Phosphacarbonyls

**Scheme 26:**
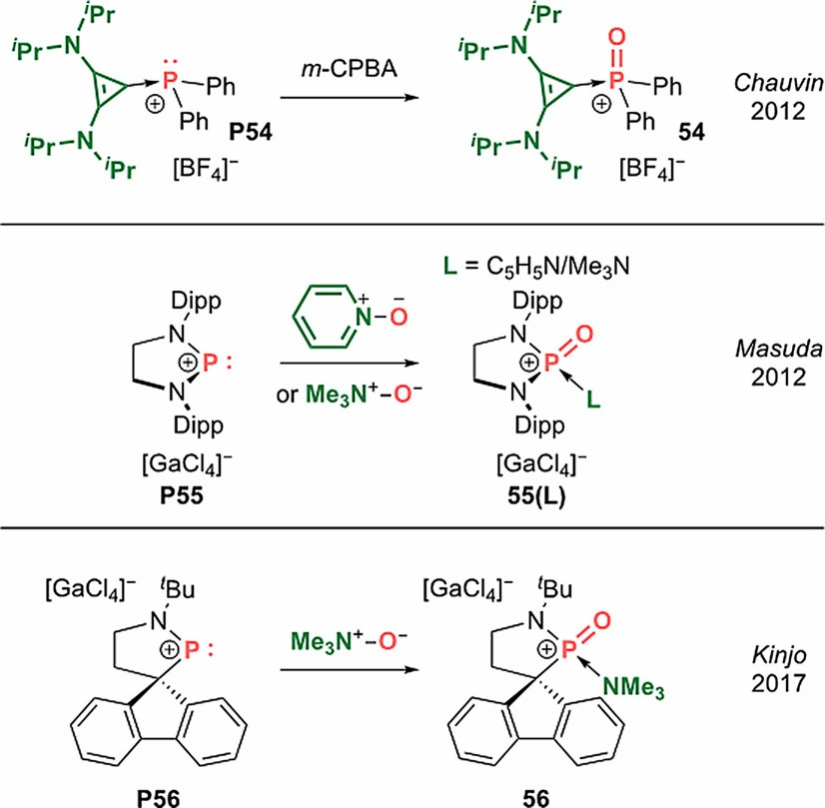
Base‐stabilized monocationic phosphacarbonyls (Type **I**).

**Monocationic analogues: [R_2_P=O]^+^ (Type I)**. In 2012, the groups of Chauvin and Masuda independently reported the synthesis of a base‐stabilized diphenyl oxophosphonium cation **54** and N‐heterocyclic oxophosphonium cations **55(L)** (L=C_5_H_5_N/Me_3_N), respectively, representing the first examples of isolable base‐stabilized phosphacarbonyls (Scheme 26, top and middle).[^44a^, ^44b^] In 2017, Kinjo et al. reported a cyclic (alkyl)(amino)phosphacarbonyl cation **56**, employing a similar synthetic route to Masuda, involving formal oxidative addition of Me_3_N^+^−O^−^ to the phosphenium precursor **P56** (Scheme 26, bottom).^[44c]^ Additional base stabilization in these systems affords tetracoordinate phosphorus centres with P=O bonds (1.4586(15)–1.4843(7) Å) which are slightly shorter than found in O=PPh_3_ (1.491(2) Å).

**Scheme 27:**
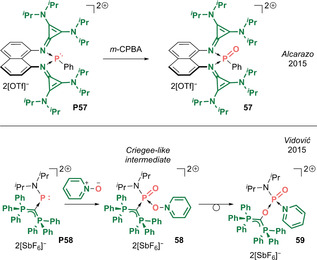
Base‐stabilized dicationic phosphacarbonyls (Type **II**).

**Dicationic analogues: [R(L)P=O]^2+^ (Type II)**. In 2015, Alcarazo et al. reported a base‐stabilized phosphacarbonyl dication **57** supported by a strongly donating cyclic bis[(dialkylamino)cyclopropenimine] ligand (Scheme 27, top).^[45a]^ In the same year, Vidović et al. reported that the oxidation of a dicoordinate phosphenium dication **P58**, supported by a strong four‐electron carbodiphosphorane donor, generated a tetracoordinate phosphacarbonyl cation **58** stabilized by an additional equivalent of C_6_H_5_N^+^−O^−^ (Scheme 27, bottom).^[45b]^ Interestingly, **58** is reminiscent of the Criegee intermediate, which has been proposed to be involved in the classical Baeyer–Villiger oxidation of ketones to esters by perbenzoic acid. Indeed, this heavier phospha‐amide rearranges to a phosphacarbamate **59** via an anti‐periplanar 1,2‐migration of the carbon group to oxygen, liberating pyridine, which then reattaches to the cationic phosphorus centre. The P=O double bond lengths measured for tetracoordinate dications (1.451(2)–1.468(1) Å) are at the shorter end associated with the tetracoordinate monocations.

### 4.3 Base‐Free Phosphacarbonyls

**Figure 7:**
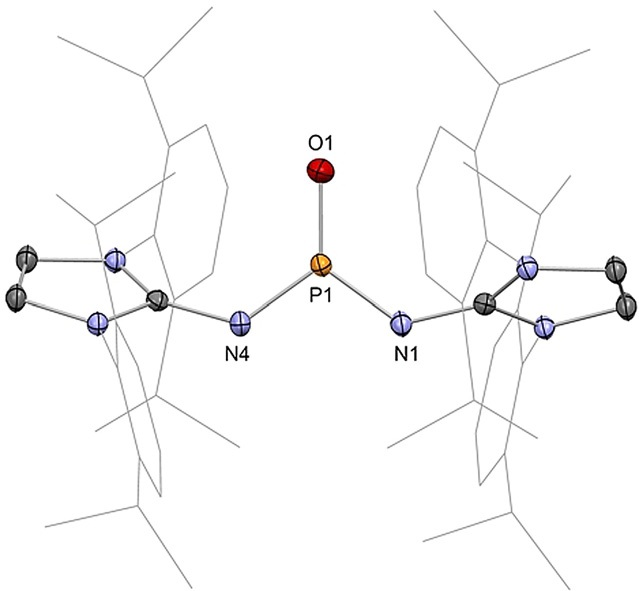
Solid‐state structure of **60(N)**. For clarity, hydrogen atoms are omitted, Dipp groups are simplified as wireframe. Thermal ellipsoids set at 50 % probability.

**Scheme 28:**
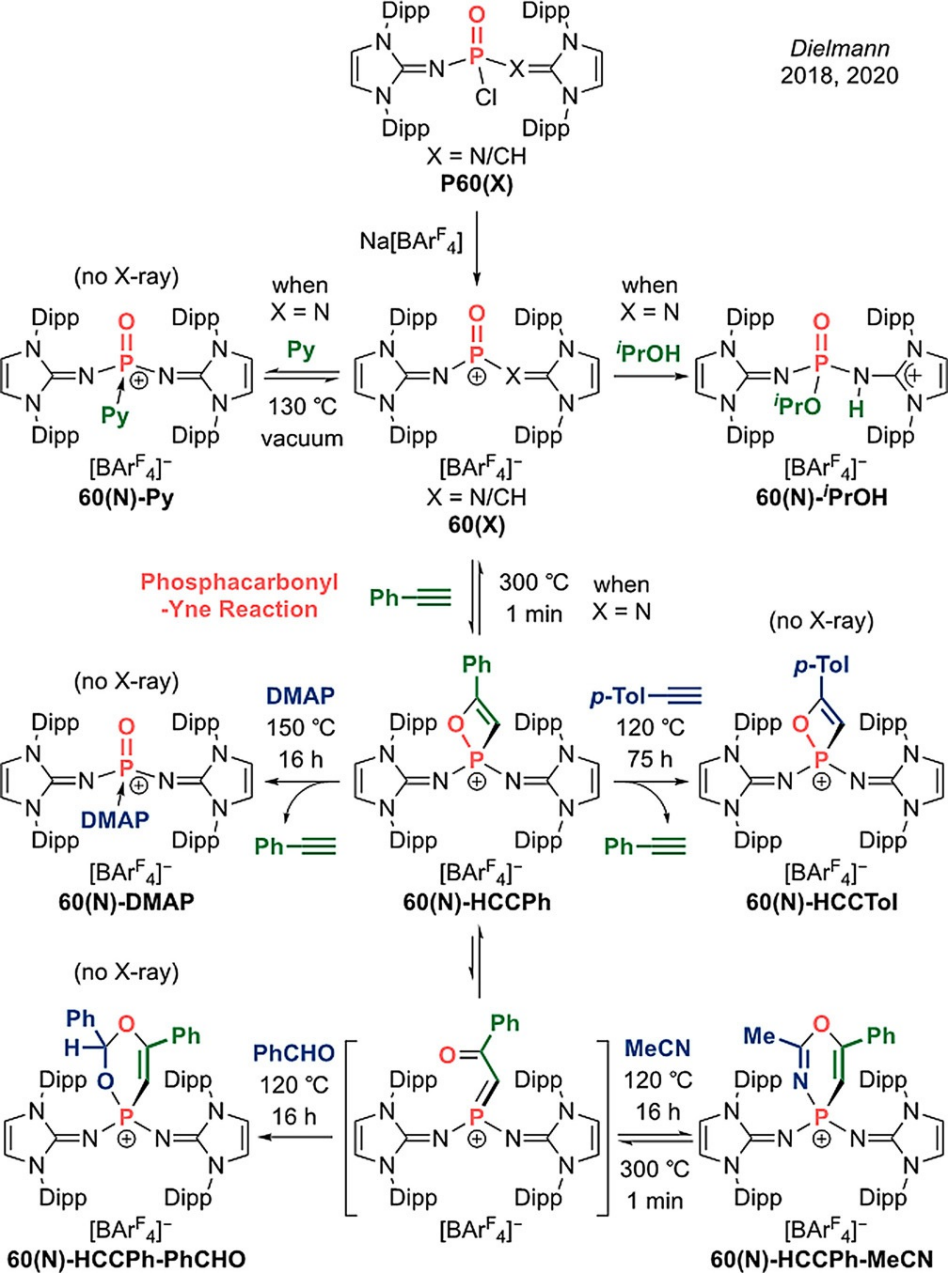
Dielmann's base‐free phosphacarbonyl **60(X)** (Type **I**) and the phosphacarbonyl–yne reaction.

In 2018, Dielmann et al. reported the breakthrough isolation of Lewis base‐free oxophosphonium monocations, which represent the first phosphacarbonyl species (Type **I**).^[11a]^ To prevent unwanted further reaction with an oxygen atom transfer agent, they devised an alternative synthetic approach via chloride abstraction from phosphoryl chlorides **P60(X)** (X=N/CH) containing a preformed P=O double bond (Scheme 28). X‐ray diffraction analysis unambiguously revealed the planar tricoordinate nature of the phosphorus centres in N,N‐phosphacarbonyl **60(N)** and N,C‐phosphacarbonyl **60(CH)** (Figure 7). The P=O bond lengths of 1.4603(9) Å and 1.463(2) Å, respectively, are in the range of base‐stabilized tetracoordinate phosphacarbonyl mono‐ and dications (**54**–**59**: 1.451(2)–1.4843(7) Å). The short P−N and P−C bonds allude to the possibility for multiple bonding between the ylidic NHI and NHO ligands, effectively acting as intramolecular electron donors to quench the strongly electrophilic nature of the central P=O moiety and removing the need for external Lewis bases. DFT analysis on the P=O fragment reveals WBIs of 1.32 for **60(N)** and 1.30 for **60(CH)** with NBO charges at P/O of +2.31/−1.03 and +2.14/−1.03 for **60(N)** and **60(CH)**, respectively. The HOMOs of **60(N)** and **60(CH)** are centred on the ylidic nitrogen and carbon, respectively, while the LUMOs correspond to the P=O π^*^ orbitals. Furthermore, the computed fluoride ion affinities (FIA) of **60(N)** (634 kJ mol^−1^) and **60(CH)** (618 kJ mol^−1^) are intermediate between B(C_6_F_5_)_3_ (425 kJ mol^−1^) and Stephan's [PF(C_6_F_5_)_3_]^+^ (795 kJ mol^−1^), hinting at their potential Lewis acidic properties. The more electrophilic nature of the bis(NHI)‐stabilized system **60(N)** than (NHI)(NHO)‐substituted **60(CH)** is reproduced with the Gutmann–Beckett method which gives an acceptor number of 102 for **60(N)**, which is in the range of Lewis superacids, while that of **60(CH)** is significantly lower at 33. Preliminary reactivity studies have also been carried out on these phosphacarbonyl species. Interestingly, **60(N)** reversibly binds pyridine, mimicking the addition–elimination mechanism of classical carbonyl compounds. In addition, **60(N)** activates ^*i*^PrOH via 1,2‐addition across the P−N bond (rather than the P=O double bond) hinting at the greater latent basicity of the ylidic ligands than the terminal oxygen atom, in a manner reminiscent of Kato's N,C‐silacarbonyl **41(** ^***i***^ **Pr)**. Overall, this work demonstrates that base‐free phosphacarbonyls are potent Lewis acids that show promise towards catalytic applications, as exemplified by the reversible binding of pyridine.

The carbonyl–yne reaction between ketones and alkynes proceeds via a [2+2] cycloaddition promoted by Lewis acid catalysis or photo‐irradiation to form intermediate oxetenes. However, the ring strain imposed by incorporation of a C=C double bond within a four‐membered ring results in subsequent collapse by electrocyclic ring opening to furnish enones. Dielmann et al. most recently reported on a heavier carbonyl–yne reaction between a base‐free N,N‐phosphacarbonyl **60(N)** and a variety of alkynes to afford isolable oxaphosphete cations (Scheme 28).^[11b]^ Notably, cyclo‐reversion is possible, evidenced by the complete liberation of phenylacetylene from oxaphosphete **60(N)‐HCCPh** at 300 °C to regenerate phosphacarbonyl **60(N)**, which could alternatively be trapped by DMAP or by exchanging phenylacetylene with 4‐ethynyltoluene. Such reversible P=O double bond formation is also reminiscent of the classical addition–elimination mechanism of carbonyl compounds. On the other hand, X‐ray diffraction analysis revealed that the P−O bond of **60(N)‐HCCPh** (1.677(2) Å) is rather long, hinting at possible electrocyclic ring opening to access a phospha‐enone cation with a P=C−C=O linkage. DFT analysis revealed the barrier to ring opening is 95 kJ mol^−1^, supporting the notion that P−O bond cleavage could be achieved at elevated temperatures. Indeed, the phospha‐enone cation could be trapped by a hetero Diels–Alder reaction with dienophiles such as acetonitrile or benzaldehyde at 120 °C to afford six‐membered **60(N)‐HCCPh‐MeCN** and **60(N)‐HCCPh‐PhCHO**, respectively, with complete scission of the P−O bond. Most remarkably, **60(N)‐HCCPh‐MeCN** is able to undergo a retro‐[4+2] cycloaddition back to oxaphosphete **60(N)‐HCCPh**, followed by a retro‐[2+2] back to phosphacarbonyl **60(N)**, with concomitant release of acetonitrile and phenylacetylene substrates. While the formation of robust P=O bonds is commonly exploited as a thermodynamic sink to drive the Wittig reaction, this work shows that a base‐free phosphacarbonyl cation can induce stepwise P=O bond cleavage (i.e. reducing the P−O bond order from 2 to 1 to 0). Most importantly, this process is entirely reversible, hence mimicking both the reversibility in carbonyl chemistry and oxide ion transfer chemistry typically associated with transition metals.

## 5 Conclusions and Outlook

Main group analogues of the ubiquitous carbonyl functional group incorporating p‐block elements have long been considered to be highly elusive entities. A major turning point in the quest for isolable main group carbonyls was the successful employment of acid/base protocols, which granted access to these transient species in their masked forms. However, the electronic and steric perturbation imposed by such chemical tricks impairs their chemical reactivity and contrasts with the rich chemistry displayed by classical carbonyl compounds.

A new era has been marked by the recent isolation of crystalline acid–base free main group carbonyl analogues ranging from a lighter boracarbonyl to the heavier silacarbonyls, phosphacarbonyls and a germacarbonyl, completely free from acid/base interference (Table 2). These synthetic achievements have been enabled by the employment of electron‐rich substituents (e.g. ylides, amino groups) with huge steric profiles, hence relinquishing the need for external acids and bases, and enabling (close‐to) unbiased comparison with classical carbonyl compounds. Most importantly, their “unmasked” nature elicits exciting new chemistry. From carbonyl‐type reactions to transition metal‐like oxide ion transfer chemistry, these systems offer to bridge the gap between carbon and transition metals, opening up to the possibility for unique “crossover” reactivity. Furthermore, the variation in overall charge from anionic group 13 to cationic group 15 main group carbonyls imbues them with additional properties as exemplified by the strong ligating abilities of boracarbonyl and potent Lewis acidity of phosphacarbonyl, while charge‐neutral silacarbonyls and germacarbonyls maintain more ambiphilic character. Hence, these main group carbonyl systems have journeyed a long way from their humble beginnings as lab curiosities, to bottleable trophy compounds, to their present status as potentially versatile reagents in chemical synthesis.

**Table 2:** Acid–base free main group carbonyls with key spectroscopic and DFT data.

Main group carbonyls	Spectroscopic data	DFT data
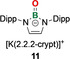	B=O: 1.273(8) Å ^11^B: 20.7 ppm	WBI: 1.40 (B=O) B (+0.99), O (−1.03)
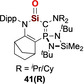	**R=** ^***i***^ **Pr** Si=O: 1.533(1) Å ^29^Si: 38.4 ppm	**R=** ^***i***^ **Pr** WBI: 1.14 (Si=O) Si (+2.16), O (−1.24)
	**R=Cy** Si=O: 1.527(3) Å ^29^Si: 40.8 ppm	**R=Cy** N.A.
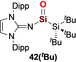	Si=O: 1.537(3) Å ^29^Si: 28.8 ppm ν_(Si=O)_: 1144 cm^−1^	WBI: 1.13 (Si=O) Si (+1.70), O (−1.23)
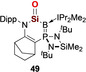	Si=O: 1.5432(12) Å ^29^Si: 71.3 ppm ν_(Si=O)_: 1130 cm^−1^	WBI: 1.09 (Si=O) Si (N.A.), O (−1.27)
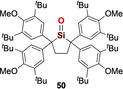	Si=O: 1.518(2) Å ^29^Si: 90.0 ppm	WBI: 1.35 (Si=O) Si (+2.08), O (−1.10)
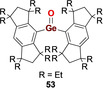	Ge=O: 1.6468(5) Å ν_(Ge=O)_: 916 cm^−1^	WBI: 1.25 (Ge=O) Ge (+1.80), O (−1.05)
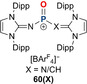	**X=N** P=O: 1.4603(9) Å ^31^P: 59.1 ppm	**X=N** WBI: 1.32 (P=O) P (+2.31), O (−1.03)
	**X=CH** P=O: 1.463(2) Å ^31^P: 99.0 ppm	**X=CH** WBI: 1.30 (P=O) P (+2.14), O (−1.03)

## Conflict of interest

The authors declare no conflict of interest.

## Biographical Information

Ying Kai Loh is originally from Singapore and is an A*STAR scholar. He obtained his BSc (Hons) in Chemistry from Nanyang Technological University (NTU) in 2015. He was awarded the Gold Medal in the Global Undergraduate Awards (2015) for research on multiply bonded main group systems. He received his DPhil in 2020 from the University of Oxford under the supervision of Prof. Simon Aldridge. He will soon embark on postdoctoral research with Prof. Guy Bertrand at the University of California, San Diego.

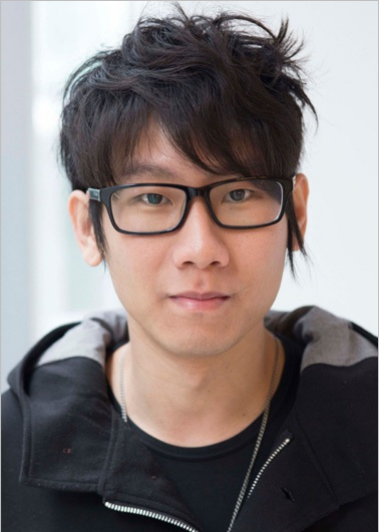

## Biographical Information

*Simon Aldridge is originally from Shrewsbury (UK) and is Professor of Chemistry at the University of Oxford, and Director of the EPSRC/Oxford Centre for Doctoral Training in Inorganic Chemistry for Future Manufacturing. He has published more than 210 papers and is a past winner of the RSC′s Main Group Chemistry (2010) and Frankland Awards (2018). His research interests include the development of p‐block compounds with unusual electronic structure and their applications in small molecule activation and catalysis*.